# A Comprehensive Review of Metabolic Dysfunction-Associated Steatotic Liver Disease: Its Mechanistic Development Focusing on Methylglyoxal and Counterbalancing Treatment Strategies

**DOI:** 10.3390/ijms26062394

**Published:** 2025-03-07

**Authors:** Izabela Berdowska, Małgorzata Matusiewicz, Izabela Fecka

**Affiliations:** 1 Department of Medical Biochemistry, Faculty of Medicine, Wroclaw Medical University, Chałubińskiego 10, 50-368 Wrocław, Poland; malgorzata.matusiewicz@umw.edu.pl; 2 Department of Pharmacognosy and Herbal Medicines, Faculty of Pharmacy, Wroclaw Medical University, Borowska 211A, 50-556 Wrocław, Poland

**Keywords:** methylglyoxal, advanced glycation end products, AGEs, AMPK, MASLD, MAFLD, NAFLD, steatohepatitis, metformin, silymarin

## Abstract

Metabolic dysfunction-associated steatotic liver disease (MASLD) is a multifactorial disorder characterized by excessive lipid accumulation in the liver which dysregulates the organ’s function. The key contributor to MASLD development is insulin resistance (IR) which affects many organs (including adipose tissue, skeletal muscles, and the liver), whereas the molecular background is associated with oxidative, nitrosative, and carbonyl stress. Among molecules responsible for carbonyl stress effects, methylglyoxal (MGO) seems to play a major pathological function. MGO—a by-product of glycolysis, fructolysis, and lipolysis (from glycerol and fatty acids-derived ketone bodies)—is implicated in hyperglycemia, hyperlipidemia, obesity, type 2 diabetes, hypertension, and cardiovascular diseases. Its causative effect in the stimulation of prooxidative and proinflammatory pathways has been well documented. Since metabolic dysregulation leading to these pathologies promotes MASLD, the role of MGO in MASLD is addressed in this review. Potential MGO participation in the mechanism of MASLD development is discussed in regard to its role in different signaling routes leading to pathological events accelerating the disorder. Moreover, treatment strategies including approved and potential therapies in MASLD are overviewed and discussed in this review. Among them, medications aimed at attenuating MGO-induced pathological processes are addressed.

## 1. Introduction

Metabolic dysfunction-associated steatotic liver disease (MASLD), previously termed non-alcoholic fatty liver disease (NAFLD) is a common disorder associated with hepatosteatosis and conditioned by systemic metabolic disturbances including metabolic syndrome and insulin resistance (IR). This review describes major aspects of the pathogenetic background of MASLD, discusses the involvement of methylglyoxal (MGO) in its development, as well as addresses therapeutic issues. The effects of poor diet, and the dysfunctions of main organs including adipose tissue (AT) and gastrointestinal tract (GIT), on the liver condition are discussed. Additionally, the impairment of cellular homeostasis contributing to MASLD is addressed. Subsequently, a putative involvement of MGO in the early MASLD stages, as well as liver cirrhosis and cancer development is discussed. A possible link between fructose oversupply, MGO, and MASLD pathogenesis is addressed. Finally, the approved and potential therapeutic modes in MASLD treatment are discussed, with respect to their potential in MGO scavenging.

## 2. Metabolic Dysfunction-Associated Steatotic Liver Disease (MASLD)

### 2.1. Current Definition of MASLD

The disease entity “non-alcoholic fatty liver disease” (“NAFLD”) characterized mainly by steatosis was renamed in 2020 into “metabolic dysfunction-associated fatty liver disease” (“MAFLD”) [1], and now (according to the current Clinical Practice Guidelines) it is termed “metabolic dysfunction-associated steatotic liver disease” (“MASLD”) [2]. The change was proposed by an international panel of experts to underline the systemic metabolic dysregulation involved in the etiology of this type of liver dysfunction. Up to 5–20% of NAFLD cases develop from simple steatosis into non-alcoholic steatohepatitis (NASH) [3,4]. In around 20–30% of NASH cases, fibrosis and cirrhosis are observed which further (in up to 2% of patients) can develop into hepatocellular carcinoma (HCC) [1,3,4,5].

Now, due to the change in the disorder term, also the subgroups of this liver dysfunction have been renamed. According to the present nomenclature: MASLD includes the condition concerning isolated liver steatosis called “metabolic dysfunction-associated steatotic liver” (“MASL”), whereas metabolic dysfunction reflected by steatohepatitis is termed “metabolic dysfunction-associated steatohepatitis” (“MASH”) [2]. Therefore, NAFLD has been renamed into MASLD, and NASH into MASH. Additionally, a new general definition of steatotic liver disease (SLD) has been coined, which encompasses liver dysfunctions associated with enhanced accumulation of lipids due to different etiologies. SLD comprises MASLD, MASLD with moderate (increased) alcohol intake (MetALD), alcohol-related liver disease (ALD), specific etiologies of SLD (e.g., drug-induced, monogenic diseases), and cryptogenic SLD [2]. The diagnosis of MASLD requires documented (by imaging or biopsy) steatosis as well as the presence of at least one cardiometabolic risk factor which includes overweight or obesity, dysglycemia or type 2 diabetes (T2DM), elevated plasma triacylglycerols (TAGs), decreased HDL-cholesterol (HDL-C), and elevated blood pressure. If additional inflammation and hepatocyte ballooning is histologically detected, then MASH is diagnosed. Since the characteristics of NAFLD are consistent with MASLD, these two terms can be used interchangeably [2].

### 2.2. The Pathogenesis of MASLD

Among the theories explaining the development of MASLD, there have been “two-hit hypothesis” and “multiple-hit hypothesis” [3]. The first hypothesis assumes that the initial pathological event (first hit) is the accumulation of lipids in the liver due to the sedentary lifestyle, high-fat diet, obesity, and insulin resistance. Subsequently, an excess of lipids primes liver cells to further pathological processes inducing inflammation and fibrogenesis—this is the second hit which promotes the progression to MASH and cirrhosis [3]. However, the multiple-hit hypothesis better describes the mechanisms responsible for this type of liver dysfunction. MASLD pathogenesis involves complex and multifactorial events both at the intracellular and systemic level. Moreover, lean individuals suffer from this disorder; hence, obesity is not a causative factor in all MASLD cases [6].

#### 2.2.1. Multiple-Hit Hypothesis

As reviewed by Buzzetti et al. [3], the leading driving force of MASLD is insulin resistance which impairs the white adipose tissue (WAT) metabolism promoting TAGs hydrolysis, thus enhancing non-esterified fatty acids (NEFAs) release from WAT to the circulation. Additionally, IR downregulates glucose (Glc) transporters GLUT-4 in the skeletal muscles and WAT which leads to hyperglycemia. These processes drive the uptake of both NEFAs and Glc by other tissues including the liver, supplying this organ with substrates for de novo lipogenesis (DNL) and TAGs synthesis. Hence, both an excessive lipids accumulation in hepatocytes as well as their secretion from the liver to the circulation in the form of very low density lipoproteins (VLDL) is observed yielding hypertriglyceridemia. Additionally, obesity and IR induce proinflammatory processes in the adipose tissue conditioned by disturbances in adipokine release, which affects the liver and other organs [7,8]. Enhanced and prolonged influx of NEFAs to the liver leads to lipotoxicity which disturbs cellular homeostasis. Consequently, intracellular organelles become impaired, especially mitochondria and endoplasmic reticulum (ER), which generates oxidative as well as ER stress [3]. Although the precise mechanism of MASLD has not been elucidated, some new findings in regard to intracellular organelle dysfunction have been recently discussed by Li et al. [6]. The authors addressed the processes induced by lipotoxicity such as ER stress which due to its perseverance in liver cells is not regulated properly by an unfolded protein response (UPR). In turn, instead of saving cells, signaling routes diverting cells into apoptosis activate. Additionally, the accumulation of lipids (such as saturated fatty acids) impairs mitochondria and lysosomes. For example, lipotoxicity-induced voltage-dependent anion channel (VDAC) increases the permeability of the outer mitochondrial membrane initiating cell death. Mitochondrial disturbances also lead to reactive oxygen species (ROS) accumulation followed by oxidative stress generation which fuels pathological processes. Moreover, lysosomal dysfunction in MASLD is probably associated with the impairment in lipophagy, which normally is responsible for the removal of lipid burdens. Finally, these organelles’ disturbances mediated by lipotoxicity-induced oxidative stress and inflammatory processes disrupt cellular homeostasis, which triggers cell death via different mechanisms (apoptosis, necroptosis, pyroptosis, and ferroptosis) [6].

#### 2.2.2. Adipose Tissue-Liver Axis

White adipose tissue seems to be the major organ responsible for the development of systemic insulin resistance which exacerbates MASLD. Unhealthy hypertrophic WAT characteristic for most obesity cases stimulates pathological processes in many organs including the liver. WAT, besides being a tissue involved in systemic energy metabolism regulation, also functions as an endocrine gland which secretes adipokines, metabolites, and extracellular vesicles (EVs) regulating a variety of processes in other organs [9,10]. EVs transport both parts of organelle as well as proteins, lipids, and different RNA forms [10]. In unhealthy obesity, the profile of secreted adipokines shifts from beneficial to detrimental. Pathological WAT secretes proinflammatory molecules (mainly monocyte chemoattractant protein 1—MCP-1 and tumor necrosis factor-α—TNF-α) which leads to the induction of low-grade inflammation and IR. This is associated with lipolysis enhancement and GLUT-4 downregulation. As mentioned earlier, such effects cause efflux of NEFAs which enter the liver contributing to lipotoxicity, as well as inhibition of Glc uptake by adipocytes and skeletal muscles, thus stimulating Glc influx to the liver. Additionally, pathologically lowered adiponectin secretion is connected with the inhibition of catabolic reactions and the stimulation of lipogenesis in the liver (e.g., through AMP-dependent protein kinase—AMPK—signaling attenuation). Some of these effects leading to IR development are mediated by faulty EVs secreted abundantly by pathological WAT [10]. They stimulate immune cells infiltrating both the WAT and the liver and promote proinflammatory cytokine production by macrophages [10]. Similar detrimental processes are observed in aging WAT whose cells undergo senescence [11]. Preadipocytes of such WAT show a decreased ability to proliferate and differentiate which impairs lipid metabolism leading to NEFAs release. Additionally, Glc transport to adipocytes is impaired which raises its blood plasma pool. Aging WAT macrophages release proinflammatory cytokines resulting in the development of low-grade inflammation. Senescence is coupled with the accumulation of dysfunctional mitochondria which generate oxidative stress and favor proinflammatory routes. Moreover, such impaired mitochondria are secreted in EVs and transferred to macrophages altering their properties which disturbs WAT metabolism [11]. Therefore, unhealthy WAT in obesity/metabolic syndrome as well as in aging stimulates pathological processes coupled with IR development and NEFAs and Glc flow into the liver, which enhances lipogenesis/steatosis and results in lipotoxicity finally impairing intracellular organelle leading to cell death. These routes are exacerbated by unhealthy WAT-released pathological profile of adipokines, metabolites, and EVs which enhance oxidative stress and low-grade inflammation in the liver.

#### 2.2.3. Hypoxia

Another pathological factor contributing to MASLD development is hypoxia associated with the activation of hypoxia-inducible factors (HIFs). HIFs alter lipid metabolism in hepatocytes diverting catabolic pathways into anabolic ones, through the regulation of a variety of genes expression. Thus, hypoxia seems to lead to TAGs accumulation, steatosis, inflammation and promotes fibrosis and carcinogenesis in MASLD [12,13,14]. In a recent report that studied mice, a high-fat high-fructose diet (HFHFD) can induce hypoxia, first in the gonadal adipose tissue, and subsequently in the liver. The hypoxia onset was accompanied by the development of early stages of MASLD characteristics (obesity, dyslipidemia, and IR without hepatitis or fibrosis occurrence). Liver hypoxia was observed only in HFHFD animals, but not in the MASH model without obesity. Therefore, this may suggest that there is a causative link between impaired WAT (hypertrophic and hypoxic) generated by an unhealthy diet (rich in Fru and fats) and the development of liver hypoxia which accelerates detrimental routes leading to MASLD development [15]. Hepatic hypoxia in MASLD seems to be connected with vascular resistance in the liver reflected by portal hypertension [16,17] and may be implicated in the development of more advanced MASLD stages (steatohepatitis and fibrosis) [15,18]. Since it was shown to be preceded by WAT hypoxia, then it might be suggested that unhealthy WAT in obesity develops hypoxia which contributes to liver steatosis through the enhanced production of NEFAs flowing into the liver [19], as well as the induction of inflammation and IR mediated by HIF upregulation [20,21]. Therefore, MASLD development is conditioned by pathological processes in other organs, and hypertrophic and/or aging WAT affected by hypoxia seems to contribute substantially to MASLD deterioration [22,23].

#### 2.2.4. Gut–Liver Axis

Other than WAT, GIT functioning exerts an important impact on the liver and its condition. The stimulatory effects of gut impairment, such as dysbiosis and increased intestine permeability, on pathological events in the liver, have been well documented [24,25,26,27]. Poor diet or overuse of antibiotics can deteriorate the microbiota profile in the GIT and increase gut permeability, thus stimulating prooxidative and proinflammatory processes in the course of MASLD. In the case of a direct link between the intestines and the liver (via the hepatic portal vein), it is widely accepted that intestinal dysbiosis is a factor influencing energetic metabolism and exacerbating metabolic, liver as well as cardiovascular disease (CVD) [26,28,29,30,31]. In MASLD, the microbiotic profile seems to be shifted towards microorganisms producing toxic/proinflammatory compounds (such as lipopolysaccharide—LPS, ethanol, phenylacetate, and branched-chain amino acids—BCAAs) at the cost of beneficial species generating short-chain fatty acids (SCFAs) including butyrate. Pathogens and toxins are transported to the liver where they bind to their respective receptors—pattern recognition receptors (PRRs). PRRs are expressed on Kupffer cells (KCs), macrophages and hepatic stellate cells (HSCs), and their induction triggers prooxidative and proinflammatory routes (via NF-κB) associated with the mobilization of T lymphocytes, neutrophils, and monocytes. These processes destroy liver tissue through the stimulation of hepatocyte death (apoptosis and necrosis) and profibrotic events (HSC activation and proliferation coupled with transforming growth factor β—TGF-β—production) [32]. Microorganism-derived toxins, except for impairing the intestinal environment (accelerating inflammatory processes and increasing permeability), also stimulate steatosis, hepatitis, and fibrosis after being transported to the liver. For example, ethanol and especially its oxidized product—acetaldehyde—may be the culprit of a variety of deleterious processes characteristic of MASLD (and resembling ALD features) [27]. Moreover, such metabolites as phenylacetate and BCAAs are able to divert metabolism into excessive lipogenesis (in the first case) or impair mitochondrial function (BCAAs), enhancing pathological processes [32]. In turn, SCFAs seem to alleviate deleterious events both in the intestine and in the liver. In GIT, SCFAs are responsible for sealing the intestinal mucosal barrier (through the stimulation of mucus production and tight junction protein expression), as well as the upregulation of anti-inflammatory regulatory T cells. In the liver, acetate, propionate, or butyrate supplementation lowers fat accumulation as well as inflammation [27,33]. These effects seem to be coupled with accelerating catabolic processes (via the induction of hepatic lipid oxidation), while attenuating lipogenic routes (via fatty acid synthase—FAS—downregulation) and proinflammatory signaling (via TNF decrease) [27]. Unfavorable microbiota profile patterns have been observed in MASLD patients, with a most firmly substantiated decrease in butyrate-producing *Ruminococcaceae* [27]. However, the cause-and-effect relationships linking dysbiosis and MASLD are not clear. Nevertheless, the manipulation of the microbiota profile promoting the growth of beneficial species (e.g., these producing SCFAs) seems to be promising in alleviating MASLD. Another approach to the improvement of microbiota profile might be the design of a prebiotic mixture which would switch the energy source for bacteria from proteins (associated with toxic products generation) to indigestible carbohydrates (prebiotics) in the distal segment of the colon [27].

Apart from the impact of end products of gut microbiota-processed dietary compounds on the liver condition, there are also reports suggesting an interplay between hepatocyte-generated bile acids and their intestinal microbial-modified derivatives in regard to the MASLD course [34]. Primary bile acids produced in the liver are transported to the intestine where they are converted into secondary bile acids by bacteria. Such a process occurs many times a day during the rounds of enterohepatic circulation. Bile acids, except for their involvement in dietary lipids emulsification in digestion, have also many functions regulating metabolic pathways through the induction of a variety of receptors [34]. Therefore, acting as signaling molecules, they trigger various intracellular pathways through binding with both intracellular receptors (e.g., farnesoid X receptor—FXR) and plasma membrane receptors (G protein-coupled receptors). Their effects exerted through FXR receptors are associated with lipid and carbohydrate metabolism regulation and are implicated in MASLD pathogenesis [27]. On the one hand, an unfavorable microbiotic profile would affect the processing of bile acids in the intestine; on the other hand, dysfunctional hepatocytes (during steatosis, hepatitis, fibrosis, and cirrhosis development) would produce altered amounts of bile acids affecting intestinal microbiota. This would lead to the vicious circle of intestinal dysbiosis accelerating hepatic dysfunction and vice versa. As reviewed by Farooqui et al. [34], different alterations in bile acid levels in MASLD (especially fibrotic) patients have been observed.

In summary, the impaired gut–liver axis, especially coupled with dysbiosis seems to be a self-accelerating mechanism leading to MASLD development. An important factor contributing to its exacerbation is an unhealthy diet.

#### 2.2.5. Dietary Fructose

A sedentary lifestyle and an unhealthy diet are among the major contributors to metabolic disorders including fatty liver. High-lipid (rich in saturated fatty acids) and carbohydrate (Glc and Fru) foodstuffs are especially associated with the increased MASLD risk. In light of the growing MASLD prevalence in children and adolescents, paralleled by the rise in sweet beverage consumption, these are high-fructose corn syrup (HFCS)-enriched meals which are especially blamed for this disorder’s increasing rates [35]. Fru and Glc contribute to liver steatosis both delivering substrates for fatty acids (FAs) and TAGs synthesis (acetyl-CoA and glycerol molecules, respectively) and inducing transcription factors (sterol regulatory element-binding protein-1c—SREBP-1c and carbohydrate-responsive element-binding protein—ChREBP) which enhance lipid synthetic pathways [35]. Fru overload seems to augment hepatic lipogenesis as well as disrupt cellular homeostasis. First, unlike Glc being metabolized in many tissues, Fru is mainly processed in the liver, where it additionally bypasses typical for Glc hormonal control (e.g., insulin/glucagon-mediated) and nutritional regulation of the rate-limiting glycolytic enzyme (phosphofructo kinase-1) [35]. Hence, Fru is uncontrollably phosphorylated resulting in the depletion of ATP which is converted into ADP and AMP, whose levels increase. Consequently, purine nucleotides undergo extensive degradation yielding uric acid. The accumulation of uric acid following fructose oversupply results in the generation of mitochondrial oxidative stress, the induction of ER stress, and the activation of SREBP-1c. These disturbances enhance lipogenesis (via acetyl-CoA carboxylase 1, fatty acid synthase, and stearoyl-CoA desaturase-1 activation) and, when they persist for a long time, promote steatosis development [35]. Therefore, Fru seems to be a more potent sugar in driving lipogenesis than Glc [36], especially in light of the recent data reported on the additional route of Fru metabolism which generates the substrate (acetyl-CoA) for de novo lipogenesis (DNL) [37]. A well-known pathway of Fru-derived acetyl-CoA in hepatocytes requires the action of ATP-citrate lyase. However, as the authors observed in their animal studies, this enzyme was unnecessary to generate acetyl-CoA from dietary Fru. This was associated with an alternative pathway of Fru catabolism. Namely, Fru was shown to be metabolized by intestinal microorganisms yielding acetate which next was transported to the liver. In the liver, it was converted into acetyl-CoA generating a substantial pool of substrates for DNL [37].

Since a fructose-rich diet and other simple sugars-rich diets can lead to addiction, as well as Fru metabolism can fuel similar pathological events as ethanol metabolism, the term “fructoholism” has been coined [38,39]. Fructoholism is understood as excessive fructose consumption which leads to psychological and physical damage and fructoholic liver disease [39]. Similar to enhanced ethanol metabolism in the liver, Fru yields intermediates which are involved in the stimulation of prooxidative (ROS generation) and proinflammatory routes (via c-jun NH2-terminal kinase-1—JNK-1) as well as lipogenic factors (SREBP-1c), which in both cases result in hepatic IR and hepatic steatosis with possible further consequences (hepatitis, cirrhosis, and CCA) [39,40]. Additionally, an excessive Fru metabolism in the intestine can impair its permeability leading to the release of endotoxins from GIT to the circulation and liver [39,41], thus exacerbating MASLD, as discussed earlier. Therefore, an excess Fru diet can impair the liver through different routes, from which Fru catabolism both in the liver and intestine (exerted by microbiota) is probably the major route in enhancing lipogenesis.

#### 2.2.6. Autophagy Versus Cell Death

Pathological processes observed in MASLD (e.g., hypoxia, oxidative stress, low-grade inflammation, and lipotoxicity) contribute to the impairment of cellular homeostasis. This disrupts intracellular organelle and their functioning. The mechanism for increasing the cell survival rates under such stressful conditions is autophagy. However, when the damages exceed the regulatory capacity of this repairing system, signaling routes divert the cell toward death.

Pro-survival autophagy seems to be impaired in a fatty liver, which is probably associated with the weakening of autophagosome fusion with the lysosome. Autophagy in hepatocytes becomes diminished along with factors predisposing of MASLD such as aging, obesity, hyperinsulinemia, and insulin resistance. Therefore, its downregulation may be implicated in human MASLD pathogenesis [42]. Especially selective autophagy types involved in damaged mitochondria removal (mitophagy) and lipid droplet degradation (lipophagy) do not seem to function properly in this disease. Mitophagy impairment is putatively responsible for the accumulation of destroyed mitochondria in fatty liver observed in MASLD. Consequently, damaged mitochondria generate oxidative stress resulting in apoptosis induction. On the other hand, properly functioning autophagy opposes routes leading to cell death stimulated by ER stress or extracellular first messengers such as cytokines. For instance, pro-apoptotic signals triggered by (TNF- or Fas-induced) death receptors are down-regulated by autophagy. Additionally, autophagy impairment may be coupled with ER stress development in MASLD since ER stress is induced by the accumulation of damaged organelles and macromolecules in the cell. Lipophagy weakening can lead to the accumulation of lipid droplets and a decrease in fatty acids beta-oxidation, thus accelerating liver steatosis. Therefore, autophagy impairment in liver cells would enhance steatosis, liver injury, and cell death [42]. Moreover, disturbances in autophagy might be involved in the acceleration of low-grade inflammation and fibrosis. This phenomenon seems to result from down-regulated autophagy in liver macrophages. This impairment might be associated with the enhancement of the proinflammatory profile of macrophages and the over-activation of macrophage inflammasomes. Eventually, it would lead to the excessive generation of cytokines (such as IL-1) and inflammation development. Attenuated macrophage autophagy might also contribute to fibrosis development. In turn, in hepatic stellate cells responsible for the induction of fibrosis, lipophagy is probably increased, which supplies these cells with substrates for energy production. In such a way, HSCs gain sufficient energy for the processes associated with their transdifferentiation into myofibroblast-like cells which excessively produce ECM components, thus generating fibrosis [42].

As previously mentioned, the accumulation of excessive stress impairs autophagy which can stimulate cell death. Different mechanisms controlled by multiple signaling pathways participate in cell death induction. Except for apoptosis, necroptosis, and pyroptosis, ferroptosis has recently gained attention as a putative mechanism responsible for liver injury in the course of MASLD [6,43,44,45,46]. Ferroptosis belongs to the regulated cell death types. It is induced by the accumulation of free iron cations and ROS, which, in the presence of polyunsaturated fatty acids, catalyze lipid peroxidation (LPO) resulting in membrane damage and cell death [47]. Iron accumulation in MASLD patients’ hepatocytes as well as enhanced ferritin levels in their serum have been observed, whereas phlebotomy seems to improve the liver condition (ameliorating fibrosis, steatosis, and hepatitis) [43,44]. A heightened iron level in MASLD patients may be associated with dysregulation of iron transport in the organism yielding an enhanced dietary iron absorption in the intestine (via upregulation of divalent metal transporter 1—DMT1) and its excessive uptake by the liver (through the upregulation of transferrin receptor 1—TfR1) [44]. Except for iron overload, MASLD patients are characterized by an increase in ferroptosis markers. For instance, LPO breakdown product—the malondialdehyde (MDA) level is increased especially in MASH patients, whereas oxidized phosphatidylcholine (which triggers ferroptosis) is detected in MASLD specimens [45]. Peleman et al. [45] proposed a model of hepatic ferroptosis in which dying cells release damage-associated molecular patterns (DAMPs) which spread deleterious processes within liver tissue, propagating pathological events characteristic of MASLD. Therefore, along with other cell death types, ferroptosis also seems to contribute to liver damage in the course of this disease.

#### 2.2.7. AMP-Activated Protein Kinase

Among a variety of intracellular regulatory enzymes and signaling pathways, AMP-activated protein kinase (AMPK) is one of the most important in regard to its involvement in MASLD pathogenesis. AMPK is an energy sensor which regulates metabolism, switching the metabolic pathways between catabolic and anabolic ones. Energy depletion activates AMPK which in turn favors processes aimed at energy production (such as mitochondrial biogenesis and FAs β-oxidation). Simultaneously, AMPK inhibits energy-requiring reactions such as DNL, cholesterol, and TAGs synthesis [48,49]. Therefore, properly working AMPK attenuates hepatic steatosis. Other than its control over the metabolism of lipids and carbohydrates, AMPK decreases the expression of proinflammatory mediators and attenuates inflammation. Namely, it inhibits signaling pathways through NF-κB and JNK routes, therefore suppressing the expression of MCP-1. Additionally, it reduces ROS generation implicated in the inflammatory processes, via the down-regulation of NOX genes and up-regulation of antioxidative enzymes (superoxide dismutase, catalase, and thioredoxin—Trx) genes. Trx up-regulation is associated with the inhibition of inflammasome activation, pointing to another anti-inflammatory effect mediated by AMPK. AMPK activity is also necessary for the prevention of hepatocyte death via apoptosis followed by liver damage. It inhibits procaspase-6 through its phosphorylation, thus stopping the proapoptotic cascade [48,50]. In turn, AMPK signaling seems to protect liver cells from lipotoxicity diverting them into autophagy [51,52]. Finally, AMPK shows antifibrotic activity attenuating TGF-β-induced expression of fibrogenic genes in hepatic stellate cells, as well as diverting the routes from HSCs proliferation and migration into the proapoptotic ones [48]. In metabolic disorders driven by overnutrition such as MASLD, AMPK is down-regulated by an excess of nutrients as well as low-grade inflammation (probably by TNFα), leading to a diminishing liver condition, whereas therapeutic effects are observed when AMPK activity is increased [48,49]. Therefore, up-regulation (restoring the proper activity level) of AMPK seems a promising therapeutic target in MASLD, especially in more advanced stages of the disorder [49,53].

#### 2.2.8. Advanced Glycation End Products

Multiple pathological processes leading to the development and exacerbation of MASLD are associated with the enhanced accumulation of advanced glycation end products (AGEs) [54,55,56]. Most commonly, AGEs are formed between carbonyl/aldehyde or ketone functional groups of reducing sugars (mainly Glc and Fru) and their trioses’ derivatives (methylglyoxal—MGO, glyoxal—GO, and glyceraldehyde—GA), and amino or guanidine residues of proteins and other compounds. Therefore, they modify their target’s structures promoting a variety of functional disturbances both intra- and extracellularly. This impairs the proper functioning of the cell, organ, and, if widespread in the whole organism, deteriorates many disorders including MASLD. Except for endogenously produced AGEs, highly processed foodstuffs can load the organism with extra AGEs disturbing the homeostasis [54,57]. Generally, AGE impact seems to be the result of their direct alteration of important macromolecules as well as their receptors-mediated signaling. Among AGE receptors, probably the most studied (as implicated in many pathologies) are RAGEs whose induction triggers different signaling pathways, often leading to ROS generation and NF-κB activation. Such routes enhance oxidative stress and an inflammatory response, being implicated in a variety of disorders. RAGE, belonging to the receptor immunoglobulin superfamily, is a pattern recognition receptor which can be induced by different ligands. It is expressed on the surface of diverse cell types, including Kupffer cells (liver-resident macrophages) and hepatic stellate cells which are involved in the stimulation of inflammation and fibrosis—the phenomena responsible for MASLD development. Apart from being embedded in the plasma membrane, RAGEs also undergo enzymatic exfoliation (yielding cRAGEs), as well as alternative splicing (releasing esRAGEs). This leads to the generation of soluble RAGE forms (sRAGEs) in the circulation, which competitively bind their respective ligands, thus attenuating the signaling effects mediated by their membranous counterparts [56]. A similar counteractive function, as compared to AGE/RAGE, is ascribed to AGE receptors 1 (AGEs-R1) which are involved in endocytosis and degradation of AGE-modified proteins, thus lowering their deleterious effects such as oxidative stress [58].

As discussed by Liu et al. [56], an accumulation of RAGE ligands (including AGEs) in the liver upregulates RAGE expression, thus accelerating oxidative stress and inflammation, and hence stimulating/exacerbating hepatic steatosis and fibrosis. For example, the induction of hepatic stellate cells’ RAGEs by (GA-derived) AGEs was shown to enhance human HSCs (LI90 cell line) activation and proliferation. These effects were mediated by the upregulation of proinflammatory (MCP-1) and profibrotic (TGF-β1, α-SMA, and collagen I) factors through the generation of ROS (produced by NADPH oxidase and electron transport chain) [59]. In turn, RAGE siRNA silencing in a rat model of (CCl_4_-induced) liver fibrosis ameliorated liver inflammation and fibrosis which was reflected by a decrease in liver damage markers (ALT, AST, ALP, bilirubin) as well as proinflammatory (IL-6 and TNFα) and profibrotic (hyaluronic acid, laminin, and procollagen type III) factors in the animals serum. Additionally, RAGE downregulation resulted in the inhibition of hepatic NF-κB, α-SMA, and collagen I expression [60]. Therefore, AGE/RAGE-induced NF-κB signaling seems to be involved in the development of liver inflammation and fibrosis. Accordingly, anti-RAGE antibodies improved liver condition (attenuating inflammation and fibrosis) and the animal’s survival in a bile duct ligation model of liver fibrosis [61]. Hence, either the reduction in AGEs and/or the downregulation of RAGEs seems to be a promising approach to alleviate MASLD. Other than limiting endogenous AGE generation (e.g., through the shift from a high sugar/fat diet to a low-calorie diet rich in fibers derived from vegetables and fruits), also highly processed foods containing AGEs should be eliminated. The reduction in dietary AGEs (dAGEs) seems reasonable in light of the experiments conducted by Leung et al. [54,57], who described the further deterioration of liver condition in MASLD rats which were fed a high dAGEs diet, in comparison with the control MASLD animals. dAGEs feeding increased hepatic AGEs and TAGs levels, oxidative stress, steatosis, steatohepatitis (CD43, IL-6, TNF α), and fibrosis (α-SMA, CTGF, collagen I). Moreover, dAGEs enhanced the activation of both HSCs and KCs which was associated with increased proliferation and oxidative stress. Additionally, HSCs demonstrated proinflammatory and profibrotic characteristics which were inhibited by NOX downregulation. Since RAGE silencing reversed these effects, they seemed to be mediated by dietary AGE stimulation of RAGEs on stellate and Kupffer cells [54,57]. Similar effects have been observed by Dehnad et al. [62] who reported upregulation of the RAGE and downregulation of AGER1 both in MASH patients and AGE-fed murine MASH model. Additionally, the authors analyzed signaling pathways which could lead to AGE induction of RAGE to AGER1 downregulation. In light of their findings, it seems that the accumulation of AGEs induces RAGE in hepatocytes which further triggers the signal transduction pathways through JNK, p38MAPK, and TGF-β and induce SMAD3. Subsequently, SMAD3 stimulates NOX4 activity which further causes degradation of Nrf2. Finally, Nrf2-mediated protection against oxidative stress development is diminished, as well as the inhibition of AGER1 expression. This leads to the acceleration of the AGE/RAGE axis stimulating proinflammatory/profibrotic processes, and the attenuation of AGE/AGER1 axis responsible for the scavenging of AGEs from the circulation. Moreover, although NADPH oxidases in hepatic stellate cells (NOX2 and NOX4) and macrophages (NOX2) contributed to the development of oxidative stress, the authors reported more eminent paracrine effects of hepatocytic AGE/RAGE stimulation (mediated by NOX4) with respect to the activation of HSCs and macrophages [62]. Therefore, it seems that the detrimental processes associated with liver inflammation and fibrosis are the result of AGE/RAGE/NOX induction in HSCs, KCs, as well as hepatocytes which, considering their prevalence in number in the liver, probably (at a certain advancement level) contribute most to these pathologies. Hence, the upregulation of extracellular AGEs’ scavenging receptors (soluble RAGE forms and AGER1) seems to be an encouraging therapeutic attitude in relieving MASLD symptoms [63].

#### 2.2.9. Hereditary Predisposition for MASLD

**Figure 1:**
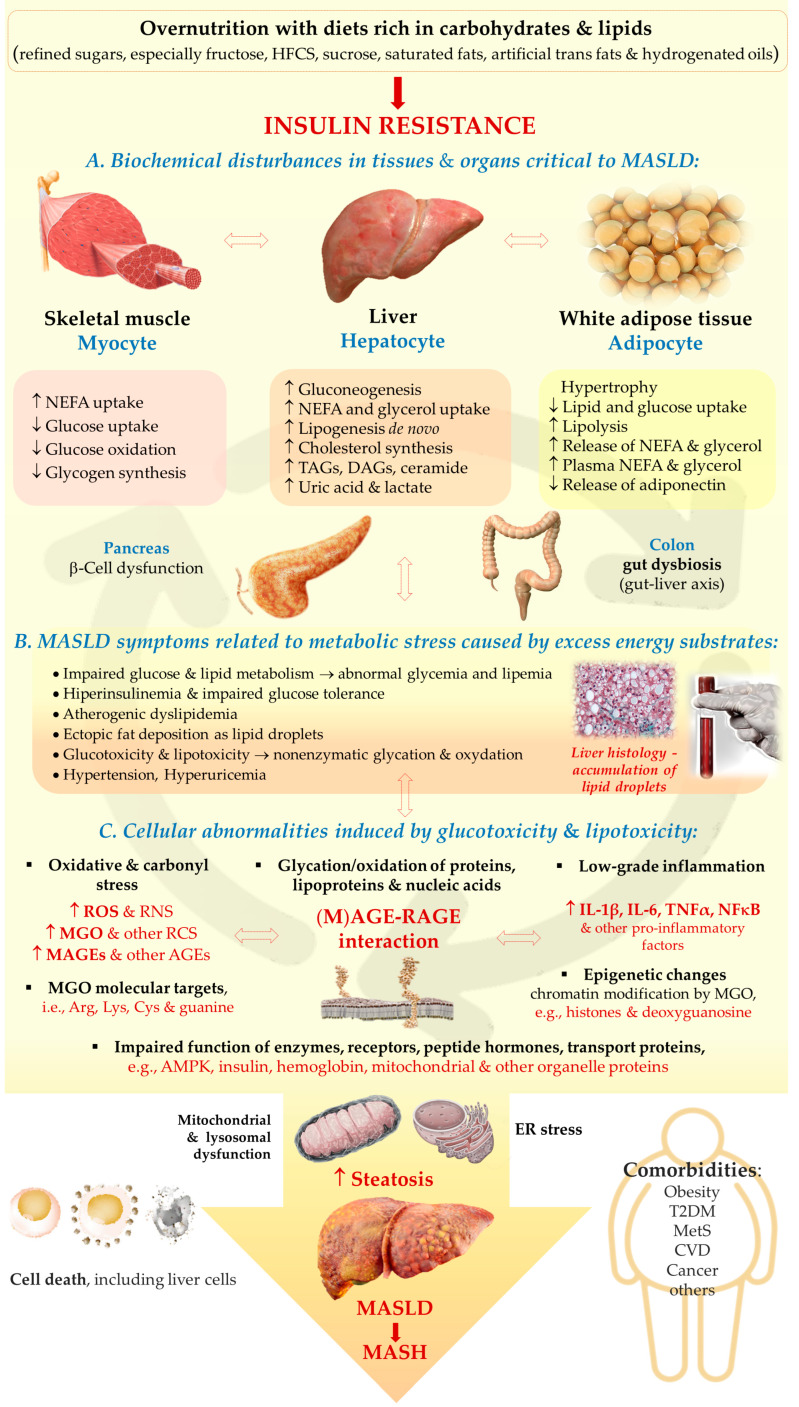
Mutually reinforcing multifactorial routes leading to the development of MASLD and comorbidities. (**A**) Poor lifestyle associated with an unhealthy diet and physical inactivity leads to insulin resistance, which impairs energy metabolism in adipose tissue, skeletal muscles, liver, and other organs. Improper diet can also lead to gut dysbiosis. (**B**) Under increased gluconeogenesis and lipogenesis, changes in the liver cause ectopic deposition of lipid droplets. Substrate stress induces glucotoxicity and lipotoxicity; it increases nonenzymatic glycation and oxidation of macromolecules. These abnormalities contribute to atherogenic dyslipidemia, impaired glucose tolerance, hyperinsulinemia, hyperuricemia, and hypertension. (**C**) A broad spectrum of advanced glycation end products (AGEs) is formed, including those derived from methylglyoxal (MGO-derived AGEs, MAGEs). (M)AGEs stimulate prooxidative and proinflammatory processes that disrupt intracellular organelles in these organs. MGO, originating from impaired carbohydrate and lipid metabolism (see Figure 2), acts either through direct damage to functional macromolecules (e.g., AMPK) or through the (M)AGEs/RAGE axis. The first mode of action causes the redirection of metabolism from catabolic to anabolic reactions, enhancing liver steatosis. AMPK downregulation also impairs autophagy and mitophagy processes, leading to mitochondrial dysfunction and cell death. (M)AGEs/RAGE axis starts from MGO modification of extracellular proteins (M)AGEs formation) which subsequently induce advanced glycation end products receptor (RAGE) signaling pathways accelerating prooxidative/proinflammatory routes. Furthermore, MGO can disturb gene expression (e.g., MGO-induced chromatin modification results in epigenetic changes). These pathological routes impair intracellular homeostasis (damage endoplasmic reticulum, lysosomes, and mitochondria), thus inducing cell death (via apoptosis, necroptosis, pyroptosis, and ferroptosis). Finally, dysfunction of the liver (and other organs) develops, progressing to steatosis, steatohepatitis, and cirrhosis. Due to the similar etiopathological background, MASLD is closely related to the coexistence of other diseases such as obesity, metabolic syndrome (MetS), type 2 diabetes mellitus (T2DM), cardiovascular disease (CVD), and cancer. These pathological events are amplified by dysbiosis and increased intestinal permeability associated with an influx of microbial toxins into the liver (gut–liver axis). Therefore, except for the bulk pool of MGO generated in the liver from an excess supply of fructose, glucose, glycerol, and fatty acids, MGO derived from the diet or gut microbiota metabolism may also contribute to the deleterious effects of this molecule in the liver; ↑, increased/intensified levels/pathways; ↓, reduced/inhibited levels/pathways.

All of the aforementioned factors implicated in MASLD pathogenesis would show a more severe impact on liver dysfunction in individuals with an unfavorable hereditary phenotype [17]. A variety of genetic polymorphisms/mutations are associated with the impairment of factors regulating lipid metabolism in the liver (de novo lipogenesis, β-oxidation, and secretion of triacylglycerols) [64]. For example, a mutated variant of transmembrane 6 superfamily member 2 gene (TM6SF2) which impairs VLDL production, enhances hepatic steatosis and fibrosis [64,65,66]. Also, a common genetic variant (rs738409) coding for patatin-like phospholipase domain-containing 3 (*PNPLA3*) is associated with fat accumulation in the liver and increases the risk of steatosis, inflammation, fibrosis, and HCC [64]. *PNPLA3* encodes adiponutrin, which is attached to lipid droplets and enables TAGs hydrolysis by lipases, whereas its mutated counterpart inhibits this process leading to fat accumulation in the liver [35]. Moreover, some epigenetic factors seem to affect gene expression in the direction of steatotic liver development including microRNA oligonucleotides (miRNAs) [3]. Therefore, MASLD development with its possible further consequences in the form of fibrosis, cirrhosis, and cancer is a multifaceted disorder both at the systemic and cellular level (Figure 1).

## 3. Methylglyoxal (MGO)

Methylglyoxal (MGO) belonging to “reactive carbonyl species” (RCS) is mainly generated as a byproduct of glycolysis and fructolysis, which is further detoxified by the glyoxalases system. Glyoxalase 1 (Glo1) catalyzes MGO transformation into lactoylglutathione—an intermediate in line to be converted into the final product D-lactate by glyoxalase 2 (Glo2) [67,68,69,70]. MGO metabolism requires a reduced form of glutathione (GSH); a co-substrate in the first reaction, which in the second reaction is regenerated [70,71]. MGO is elevated in metabolic disturbances associated with hyperglycemia and hyperlipidemia such as metabolic syndrome (MetS), T2DM, and CVD where it participates in MGO-AGEs (MAGEs) formation. This is associated with the destruction of macromolecules (proteins, lipoproteins, and DNA) which impairs intracellular organelle (e.g., mitochondria) as well as extracellular matrix (ECM) functioning. Some of the deleterious effects are mediated by AGE receptors (RAGEs) whose induction stimulates prooxidative and proinflammatory pathways underlying metabolic disturbances (recently reviewed in [72]).

Except for glyoxalases, DJ-1 might be implicated in protection from carbonyl stress decreasing MGO-induced glycation of macromolecules [73,74,75]. DJ-1 is a multifunctional protein which acts as a sensor for cellular oxidative stress, in response to which it turns on protective mechanisms through several signaling pathways [76]; additionally, it controls the activity of mitochondria (being engaged in mitophagy) [77]. DJ-1 seems to show glyoxalase and deglycase activities [73,74,75]. However, its function in MGO detoxification has been challenged in studies based on fruit flies and mammalian experimental models [78,79]. As observed by Mazza et al. [79], DJ-1 actually shows glyoxalase activity, but it is much weaker in comparison to Glo1, so its significance in vivo is questionable (but might be compensative, e.g., during GSH depletion). DJ-1 deglycase activity is more controversial with some studies supporting it [74,75,80,81], whereas others show contradictory results [78,79,82].

**Figure 2:**
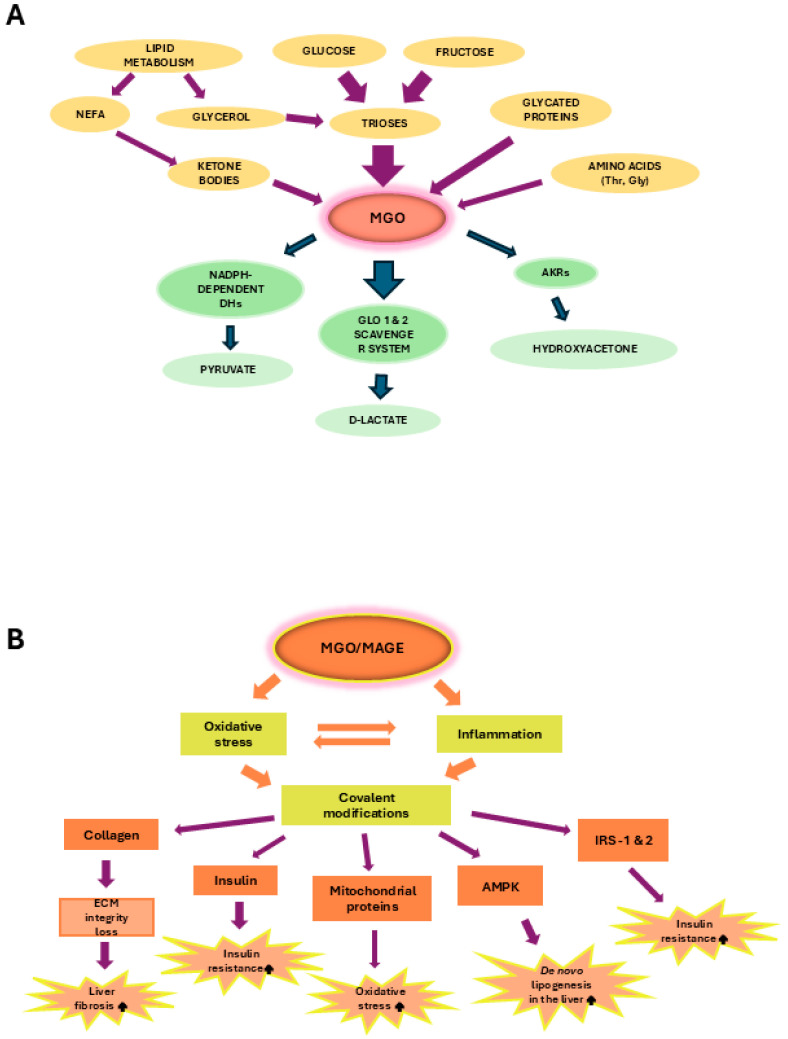
(**A**) The sources and fates of methylglyoxal (MGO). The main route of MGO generation and metabolism is shown in the center, visualized by the widest arrows. Glucose and fructose metabolites are the major MGO source, and glyoxalases 1 and 2 (Glo1 and Glo2) comprise the main MGO scavenging system. However, in metabolic disturbances initiated by insulin resistance, lipid sources probably contribute significantly to the hepatic MGO generation. They include glycerol (excessively entering the liver due to enhanced lipolysis in white adipose tissue—WAT), as well as (keto)aldehydes—products of lipid peroxidation which increase due to oxidative stress. Also, the activity of glyoxalases, sometimes impaired in pathological conditions, might be compensated by NADPH-dependent dehydrogenases (NADPH-dependent DHs), as well as aldoketo reductases (AKRs). Substrates leading to MGO synthesis are shown in yellow. Enzymatic systems involved in MGO scavenging and the final products of MGO metabolism are depicted in green. (**B**) Detrimental effects of excessively generated MGO and its advanced glycation end products (MAGE). MGO can directly lead to oxidative stress development which accelerates inflammation. Additionally, MGO modifies different macromolecules impairing their functionality. Such disturbances can result in the development of insulin resistance (disruption of insulin and its signaling), liver fibrosis (collagen damage), or de novo lipogenesis stimulation (via AMPK inhibition). IRS-1 and -2 are insulin receptor substrates 1 and 2.

The catabolism of Glc and Fru is the main source of MGO, and Glo1/2 system is a major MGO scavenger. However, in (physio)pathological processes other molecules can contribute to MGO generation and additional enzymes can promote metabolization. Minor MGO sources comprise amino acids, glycerol, ketone bodies, aldehydes, and ketoaldehydes generated from lipid peroxidation, as well as glycated proteins [67,69,83,84,85]. These molecules can contribute more to MGO generation, especially in metabolic disturbances, during which an excessive accumulation of MGO can result both from an accelerated glycolytic/fructolytic flux, but also from lipid peroxidation or glycerol. The first route is fueled by an excessive Glc and Fru supply (in a sugar-rich diet, obesity, metabolic syndrome, and T2DM). The second pathway is augmented in hepatic steatosis and accelerated by insulin resistance—driven by elevated NEFAs and glycerol flux into the liver (Figure 2). Since metabolic dysfunctions underlying MASLD, usually both routes are enhanced, therefore multiple MGO sources lead to this molecule over-production. Although MGO scavenging is mainly conducted by the glyoxalases system (which metabolizes above 98% of this molecule), other enzymes can promote degradation. These include NADPH-dependent aldehyde dehydrogenases—ALDHs—which convert MGO into pyruvate, and aldoketo reductases—AKRs—metabolizing MGO into hydroxyacetone [67]. These extra MGO-scavenging enzymes may play a compensatory function in protecting from enhanced glycation, especially in pathological processes coupled with Glo1/2 downregulation [70,86,87]. The main aspects of MGO metabolism are summarized in Figure 2A.

The most common MGO-generated AGEs (MAGEs) are protein arginine (Arg) modifications resulting in hydroimidazolones generation. The major hydroimidazolone is MG-H1 (but MG-H2 and 3 isoforms also occur) [67,88,89,90]. Additionally, Arg can be converted into tetrahydropyrimidine (THP) or argpyrimidine (ArgP) by MGO [91,92]. Other amino acid residues modified by MGO include lysine (Lys) or cysteine (Cys) side chains [93]. Lys modification yields a carboxyethyl derivative (CEL) [67]. As discussed in a previous review paper [72], a variety of structurally and functionally important proteins can undergo MGO-glycation, which can lead to intra- and extracellular disturbances. They include albumin, hemoglobin, insulin, collagen, histones, and mitochondrial proteins. For example, MAGEs can disrupt the protein’s structure to enhance their resistance to degradation and/or impair proteasomal systems to limit the proteolytic capacity of the cell [67,72]. Such phenomena might lead to the accumulation of misfolded proteins and the activation of ER stress, which if exacerbated can cause cell death via different mechanisms. The implication of ER stress in MASLD has been recently reviewed [94,95]. MGO-modified insulin may attenuate an enhanced (by normal insulin) Glc uptake by muscles and adipose tissue, hence insulin-resistance (being involved in MASLD) can be stimulated. Collagen modification by MGO can impair extracellular matrix (ECM) integrity and enhance fibrosis in liver disease. MGO’s effect on mitochondrial proteins can lead to mitochondrial dysfunction stimulating oxidative stress (being one of the pathological processes observed in MASLD). Additionally, as proposed by Gugliucci et al. [96], MGO may modify AMPK, impairing this kinase activity, which leads to the switching of metabolic pathways from catabolic to anabolic, enhancing FAs and TAGs synthesis in the liver (observed in MetS and MASLD). Some of the MAGEs effects can lead through the alteration of gene expression, either directly by nucleic acids glycation (mainly forming CEdG and MG-dG derivatives of deoxyguanosine [67,70]), or via epigenetic regulation (through the modification of Arg and Lys residues on histones [73]). Therefore, a lot of MGO/MAGEs-induced routes leading to pathological processes are possible which accelerate the development of MASLD (Figure 2B).

## 4. MGO in MASLD

Methylglyoxal has been associated with the development and progression of many pathological processes such as systemic insulin resistance which is a feature of metabolic syndrome and type 2 diabetes as we previously reported [72]. The pathological changes leading to IR result either from aberrant insulin signaling pathways observed in this condition or from changes in the structure of the insulin molecule itself that may be procured by MGO action. The background and factors accompanying MASLD and cardiovascular disturbances are interwoven with each other and these conditions often coexist. MGO involvement in MASLD-associated pathologies or comorbidities such as oxidative stress, low-grade inflammation, obesity, hypertension, (proatherogenic) dyslipidemia, and hyperglycemia (prediabetes/T2DM), has been addressed in an earlier publication [72]. Therefore, in the present review, we focus on the pathological events observed in the liver (and reflected in biological fluids) which may be mediated by MGO.

### 4.1. MGO in the Early MASLD

**Figure 3:**
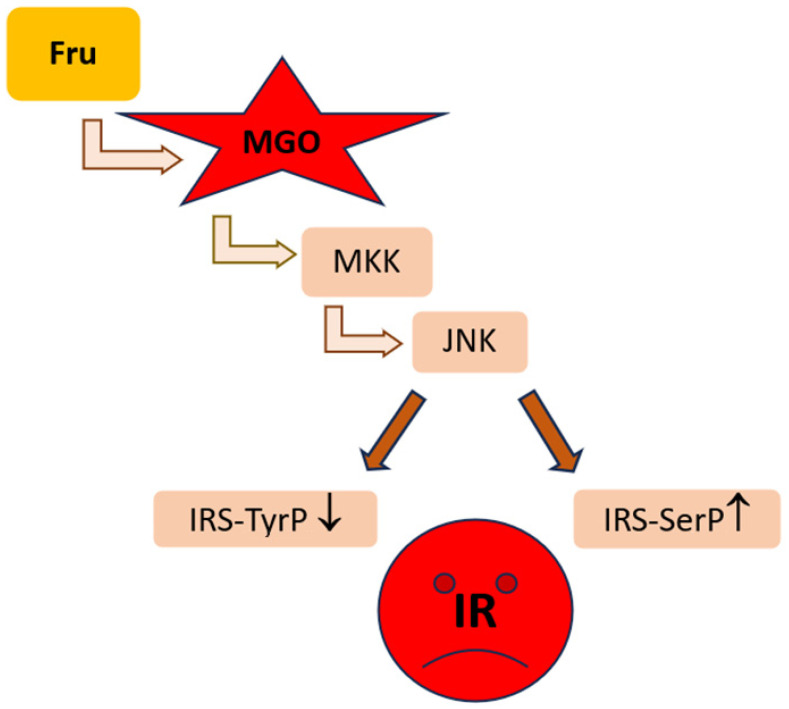
A possible pathway of insulin resistance induction by Fru-derived MGO in the liver [98]. MGO accumulation in hepatocytes causes activation of kinases cascade which leads to the inhibition of tyrosine phosphorylation on an insulin receptor substrate paralleled by enhanced serine phosphorylation. This impairs insulin-triggered signaling cascade which results in insulin resistance development. MKK7, mitogen-activated protein kinase kinase 7; JNK, c-jun NH2-terminal kinase; IRS-TyrP↓, decrease in insulin receptor substrate phosphorylation at tyrosine; IRS-SerP↑, increase in insulin receptor substrate phosphorylation at serine; IR, insulin resistance.

Among MASLD patients, the majority show the features of simple steatosis; however, only in a minority of cases does the disease develop into steatohepatitis and may progress into cirrhosis and cancer [3,4]. The initial stages of MASLD are associated with the development of insulin-resistance, the accumulation of FAs/TAGs in the liver, and ballooning/low-grade inflammation. In an animal model of early MASLD (CCl_4_-treated rats showing some hepatic steatosis, apoptosis, and ballooning, but no portal inflammation and fibrosis) [97], MGO concentration was elevated in the liver; however, this change was not reflected by the MGO level in the serum nor in the urine. Instead, a hepatic MGO increase was paralleled by the rise in its detoxification product D-lactate both in the liver and serum, as well as D-lactate urinary excretion was enhanced, in the fourth week of CCl_4_ treatment. Since the liver-damage markers were not enhanced in the serum, these findings suggest MGO implication in the early stages of MASLD, which precede more severe liver destruction [97] (Table 1A). MGO involvement in the development of insulin resistance in hepatocytes has been postulated by Wei et al. [98]. In their experiments on rat hepatocytes, the authors observed that MGO disturbed insulin signaling components in an analogical way as Fru. Based on their findings, the authors concluded that detrimental actions of Fru excess were mediated by the increase in MGO in hepatocytes. These effects included the attenuation of insulin signaling through the inhibition of tyrosine phosphorylation on insulin receptor substrates (IRS-1 and 2), accompanied by the activation of serine^307^ phosphorylation on IRS-1 being a consequence of JNK activation [98,99]. Accordingly, MGO-mediated Fru influence on hepatocytes was shown to stimulate signaling routes associated with the activation of MKK7 and JNK kinases [98] (Table 1A). Therefore, since MKK7 is an activator of JNK [100], Fru-high foodstuffs can induce the processes leading to IR in the liver through the elevation of MGO which in some way leads to the induction of signal transduction cascade activating MKK7, and subsequently stimulates JNK. Finally, the inhibition of insulin signaling via the change in the phosphorylation pattern of IRS is observed (Figure 3).

To assess MGO and MAGE implication in MASLD induction, an animal model with diet-induced obesity and methylglyoxal-induced glycation was reported by Neves et al. [101]. MGO supplementation of rats fed a high-fat diet (HFD) led to the initiation of the events characteristic for MASLD onset. Namely, MGO treatment caused the accumulation of its specific glycation products in the liver. This was associated with oxidative stress and infiltration of the liver with inflammatory cells (especially in portal regions), as well as disturbances in lipid metabolism. MGO/MAGEs resulted in the increase in NEFAs accompanied by the attenuation of (HFD-stimulated) TAGs generation in the liver, which led to lipotoxicity. NEFAs were elevated in the blood plasma of HFD/MGO-treated rats, whereas in their livers this treatment lowered the level of glycerol esterification with FAs as well as altered the profile of saturated vs. unsaturated FAs in favor of the saturated ones. Except for decreasing TAGs synthesis, MGO treatment enhanced the synthesis of FAs through the upregulation of FAS and attenuation of ACC inhibition by HFD [101] (Table 1A). Some effects could be mediated by MGO/MAGE-caused inhibition of AMPK signaling. Silencing of AMPK routes either directly (via MGO-modified functional Arg residues in AMPK [96]) or indirectly (by MGO-caused lowering adiponectin) would switch metabolic routes from catabolic into anabolic ones. As a consequence, lipogenesis instead of FA β-oxidation was accelerated. Additionally, insulin-resistance and dysglycemia features were observed upon MGO stimulation. They included hyperinsulinemia, increased HOMA values, and enhanced Glc intolerance (AUC elevation), which were raised by an HFD adiponectin concentration decrease upon MGO treatment. Additionally, MGO/MAGEs probably inhibited insulin signaling and Glc uptake by hepatocytes diminishing the activation of insulin receptors and downregulating Glc transporters GLUT-2 whose expression rose upon HFD [101] (Table 1A).

Peter et al. [102] studied non-pathological liver fat from liver biopsies performed due to the resection of solitary liver lesions. The BMI of patients in the studied cohort ranged from 24.6 to 29.8 kg/m^2^ (average 26.6 kg/m^2^). They observed that even at such an early stage, with upper range or slightly elevated BMI, MASLD could potentially be developed. Glo1 activity reflected body fat content, inversely correlating with TAGs. Glo1 activity also decreased alongside the HOMA increase, already reflecting changes in cells susceptibility to insulin at this stage. Interestingly, they did not observe associations between Glo1 activity and dicarbonyl compounds (MGO, glyoxal) elevation or glycation indices. They concluded that Glo1 activity could be useful as a prognostic marker of MASLD. They observed sex dimorphism in protein expression as well as the activity of Glo1, which may impact future studies and conclusions. Both of these factors were lower in females than males. At the same time, there were no significant differences in MGO concentrations between sexes. Downregulation of Glo1 in females may imply that they may be more influenced by the effects of high levels of MGO and its AGEs than males.

Pathological features characteristic for early MASLD, such as oxidative stress and TAGs increase, have been associated with MGO accumulation in the liver, as observed in animal models (hereditary hypertriglyceridemic male rats—HHTg, and female rats with postmenopausal MetS) [103,104] (Table 1A). In HHTg, the application of salsalate (salicylate ester of salicylic acid) led to an increase in Glo1 which downregulated MGO and improved liver condition [103]; however, in the second model, MGO increased due to ovariectomy being not coupled with the change in Glo1 expression [104]. Nevertheless, the stimulation of Glo1 expression, which would scavenge MGO and attenuate its unfavorable actions, seems a promising strategy for ameliorating MASLD. Such an effect has been shown to result from genistein (soybean isoflavone) application in a murine model of metabolic syndrome, where upregulation of Glo1, Glo2, and aldose reductase in the liver and kidney was coupled with lowering MGO and AGEs levels in blood plasma. Additionally, genistein diminished AGE generation in the liver and kidney. Except for the influence on MGO scavenging enzymes, the authors reported other mechanisms of MGO removal. Namely, the formation of adducts between genistein and MGO followed by their excretion from the organism in urine was reported. Moreover, as shown in this work, the downregulation of RAGE by genistein may also contribute to the attenuation of MGO deleterious effects exerted through these receptors induction [105] (Table 1A). A different animal model of MASLD was reported by Spanos et al. [106], who compared normal and apolipoprotein E knockout (ApoE^−/−^) mice fed a normal (ND) or high-fat (HFD) diet. After 12 and 16 weeks of HFD feeding ApoE^−/−^ mice developed minimal to mild steatosis and showed significantly decreased expression of Glo1 in comparison with upregulated Glo1 in ApoE^−/−^ mice on a normal diet. Also, FAs treatment of hepatoma cells (HepG2), which led to lipids accumulation in the cells, downregulated Glo1 and elevated MGO levels [106]. The insight into the mechanism pointed towards Glo1 hyperacetylation followed by ubiquitination and finally proteasomal degradation of the enzymatic molecule [106]. Hence, due to lipotoxicity-induced insufficient scavenging capacity, this would lead to the accumulation of MGO in hepatocytes as well as its release to the extracellular environment, with further consequences in the form of carbonyl stress and MAGE formation in the liver. Disturbances in MGO metabolism in a murine MASH model have also been observed in the HFD fed LDLR^−/−^ mice (C57BL/6J) [107]. The animals’ group which developed hepatosteatosis, hepatic damage, inflammation, oxidative stress and fibrosis, also showed lowered levels of S-lactoylglutathione (an intermediate in MGO metabolism produced by Glo1) in the liver. However, neither Glo1 nor Glo2 mRNA levels were affected by diet [107].

**Figure 4:**
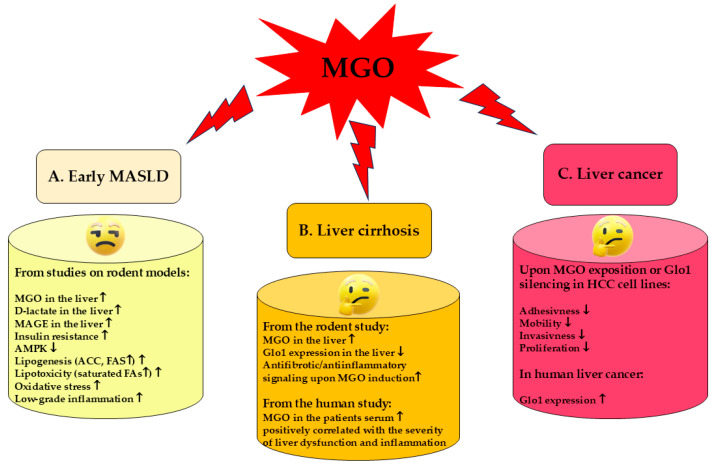
A summary of the effects associated with MGO involvement in early MASLD [97,98,101,103,104,105], liver cirrhosis [108,112], and liver cancer [109,110,111]. The picture is based on data thoroughly discussed in Section 4 and accumulated in Table 1. Whereas a detrimental MGO impact in early MASLD is well documented, its function in liver cirrhosis and liver cancer is not clear due to the scarcity of available data (as discussed in Section 4). ↑, upregulation; ↓, downregulation.

In light of these findings, MGO is implicated in the development of MASLD, since its concentration in the liver increases in rodent models showing features of this disorder [97,103,104]. MGO accumulation in the liver parallels oxidative stress generation and lipogenesis. Additionally, rodent and cell line experiments (based on MGO or Fru supplementation) point to MGO as a factor contributing to insulin resistance development in the liver through the inhibitory effect on insulin receptor [101] and insulin receptor substrate (IRS) [98]—two main initial components in insulin-induced signaling pathway. MGO accumulation in the liver can be associated with its accelerated synthesis caused by the oversupply of lipid and carbohydrate substrates (Figure 2), but also with the impairment of MGO metabolism connected with the increased degradation of Glo1 stimulated by its (FAs-fueled) hyperacetylation [106]. TAGs accumulation is a major feature which leads to hepatosteatosis in MASLD, and accompanies MGO increase [103,104]. However, MGO is involved in the stimulation of DNL and favoritism of saturated FA generation, whereas its impact on TAGs synthesis was shown to be inhibitory [101]. Therefore, MGO accumulation is probably responsible for the generation of FAs and its toxic products which further contribute to detrimental effects on cell membranes, intracellular organelles, and ECM components. MGO impact in early MASLD is summarized in Figure 4A.

**Table 1:** Methylglyoxal and glyoxalase 1 in MASLD.

Experimental Model	Detailed Observations	Major Findings in the Liver (Tissue/Cells)	Ref. Year
A. Early MASLD			
The model of early MASLD: seven-week-old male Wistar rats (WRs) divided into 2 groups: (1) WR injected with (0.3 mL/kg/week of 40%) CCl_4_ (in soy bean oil) for 4 weeks. (2) WR injected with the same volume of soybean oil (control group)	Serum (as compared with control group): D-lactate ↑; AST, ALT and MGO =. Liver: MGO level and D-lactate ↑. Urine: D-lactate ↑; MGO =.	MGO ↑ D-lactate ↑	[97] 2018
The model of hypertriglyceridemia/prediabetes: (1) Six-month-old male hereditary hypertriglyceridemic rats (HHTg) as the non-obese prediabetic model treated or not-treated with salsalate (2) WR as the control group	In HHTg rats (in comparison with WR and attenuated by salsalate); Liver: TAGs, Chol and MGO ↑; oxidative stress ↑ (TBARS ↑, GSH/GSSG ↓, SOD ↓). Upon salsalate treatment in HHTg: *Glo1* gene ↑ associated with MGO ↓.	MGO ↑ Lipids ↑ oxidative stress ↑	[103] 2023
The model of postmenopausal MetS: Female Wistar rats (WRs) divided into 2 groups: (1) Ovariectomized WR used as a model of postmenopausal MetS (W-OVX); (2) Sham-operated WR as a control (W-sham)	In W-OVX rats (in comparison with W-sham rats); Serum: leptin, FAs, HDL-Chol, MCP-1 ↑; TAGs and Chol =. Liver: MGO and TAGs ↑; Glo1 (mRNA and activity) and Chol =; oxidative stress ↑ (TBARS ↑, GSH/GSSG ↓, GPx ↓); Muscle: TAGs ↑.	MGO ↑ TAGs ↑ oxidative stress ↑	[104] 2021
The model of MGO-enriched high-fat diet: Male WR divided into four groups: (1) control (Ct) with standard diet A03 (5% triglycerides and 45% carbohydrates) (2) methylglyoxal group (MG) with a standard diet and MGO administration (rats fed 75 mg MGO kg-1 daily for 18 weeks) (3) high-fat diet-fed group (HFD) (40% triglycerides and 10% carbohydrates) (4) high-fat diet group with MGO supplementation (rats fed 75 mg MGO kg-1 daily for 18 weeks) (HFDMG)	Effect of MGO supplementation (HFDMG group compared to control and/or MG or HFD rats); Blood plasma: NEFAs ↑; albumin ↓; adiponectin ↓ (as compared to adiponectin ↑ in HFD). Liver: Inflammatory cells ↑ (F4/80 ↑—a marker of macrophages/Kupffer cells); MAGEs ↑ (MG-H1 ↑, CEL ↑, but ArgP =); Insulin receptor phosphorylation at Tyr1163 ↓; Phosphorylation of ACC ↓ (ACC activity ↑); Phosphorylation of AMPK ↓ (AMPK activity ↓) Cardiolipin 70:2 ↓; Expression of FAS ↑ and AceCS ↑; membrane RAGE =; Glo1 expression = (but Glo1 activity ↑ in MG; Glo1 activity ↓ in HFDMG)	Inflammation ↑ MAGE ↑ IR ↑ ACC ↑ AMPK ↓	[101] 2019
The model of genistein effect evaluation in high-fat diet: Male C57BL/6J mice divided into 8 groups: Study 1 (mice fed for 16 weeks with): (1) low-fat diet (10% fat energy) (LF) (2) very-high-fat diet (60% fat energy) (VHF) (3) very-high-fat diet with 0.25% genistein (VHF-G). Study 2 (mice fed for 18 weeks with): (4) low-fat diet (10% fat energy) (LF) (5) moderately high-fat diet (HF) (6) moderately high-fat diet with MGO (110–145 mg/kg/day) (HFM) (7) moderately high-fat diet with MGO and 0.067% genistein (HFM-GL) (8) moderately high-fat diet with MGO and 0.2% genistein (HFM-GH)	Genistein effect (VHF-G vs. VHF and HFM-GH vs. HFM); Blood plasma: MGO ↓, AGEs ↓, Glc ↓, Chol ↓, ALT ↓, AST ↓. Liver and kidney: AGEs ↓; Glo1/2 expression ↑, aldose reductase expression ↑; RAGE expression ↓. Liver: TAGs level ↓.	Glo1/2 ↑ TAGs ↓ RAGE↓ AGEs ↓	[105] 2019
Fru/MGO effect on rat hepatocytes: (1) Primary rat hepatocytes (isolated from WR) (PRH) incubated with Glc (8 mM) and inulin (0.12%) with or without inulinase in the absence or presence of insulin for up to 4 h. (2) PRH incubated with Glc (8 mM) and inulin (0.12%) and MGO (20 µM) in the absence or presence of insulin for 4 h.	Effect of Fru on PRH (in comparison with Glc-exposed PRH): MGO ↑ (~ 2-fold). Effects of Fru or MGO on PRH: phosphorylation of MKK7 ↑; phosphorylation of JNK ↑; phosphorylation of serine^307^ on IRS-1 ↑ (in the absence and presence of insulin); insulin-stimulated tyrosine phosphorylation of IRS-1 and IRS-2 ↓.	Fru effect: MGO ↑ Fru/MGO effect: IR ↑	[98] 2013
B. Liver cirrhosis			
The model of liver cirrhosis: (1) Male WR treated with CCl_4_ and phenobarbital for 8 weeks (early cirrhosis without ascites) or 12–14 weeks (advanced cirrhosis with ascites) (2) Male WR treated with CCl_4_ for 12–14 weeks, and Glo1 inhibitor (ethyl pyruvate—EP) starting from week 8. Primary rat hepatocytes (pHEP), primary hepatic stellate cells (pHSC) and primary liver sinusoidal endothelial cells (pLSEC) isolated from control and cirrhotic WR. Normal hepatic stellate cells (HSZ-B-S1).	In comparison with pHEP: Glo1 expression in pHSC and pLSEC derived from control WR ↓. In the whole liver, and pHEP, pHSC, and pLSEC in cirrhosis (in comparison with healthy WR): Glo1 expression ↓ (and lower in advanced cirrhosis as compared to early cirrhosis). In pHSC and pLSEC in cirrhosis (in comparison with healthy WR): Glo1 activity ↓. In the whole liver and pHEP in cirrhosis (in comparison with healthy WR): Glo1 activity ↑ In the whole liver in cirrhosis (in comparison with healthy WR): MGO level ↑ (and higher increase in advanced cirrhosis as compared to early cirrhosis) Upon LPS induction of HSZ-B-S1: Glo1 activity ↑. Upon EP or MGO treatment of LPS-induced HSZ-B-S1: TNF-α ↓, collagen-I ↓, α-SMA ↓. Upon EP treatment of LPS-induced HSZ-B-S1: LPS-induced NF-κB stimulation ↓, LPS-induced reduction in Nrf2 ↓, LPS-induced pERK ↓, ERK expression =. Effect of EP treatment on cirrhotic WR (compared to cirrhotic livers without EP treatment): fibrotic tissue ↓, α-SMA ↓, TGF-β ↓, NF-κB expression ↓, Nrf2 expression ↑.	MGO ↑ Glo1 expression ↓ Glo1 activity ↑ (liver and hepatocytes) Glo1 activity ↓ (pHSC and pLSEC)	[108] 2017
C. Hepatocellular carcinoma			
Human HCC cell lines: Huh-7, HepG2 and Hep3B.	Effect of 1 µM MGO on Huh-7 and HepG2 cells (but not Hep3B): cells adhesion to collagen ↓, cells invasion through Matrigel ↓ (via promoting p53 localization in the nucleus).	MGO effect: invasiveness ↓	[109] 2013
Human HCC cell lines: Hep3B, SK-HEP-1 and SMMC-7721	Effect of Glo1 knock-down in all 3 cell lines: cells proliferation ↓. Effect of Glo1 over-expression in all 3 cell lines: cells growth =.	Glo1 silencing effect: proliferation ↓	[110] 2014
Human HCC cell lines: Huh-7 and HepG2 Murine hepatocyte cell line AML12	In comparison with normal AML12 cells: Glo1 in Huh-7 ↑ (mRNA, protein and activity); Glo1 in HepG2 ↑ (only mRNA); Effects of Glo1 inhibition in Huh-7 cells (by 1–20 mM ethyl pyruvate or 1–10 µM BrBzGSHCp2): proliferation ↓, migration ↓, colony formation ↓; PDGFR-β ↓, VEGFR2 ↓, VEGF ↓, pERK/ERK ↓, NF-κB ↓; Nrf2 ↑. Effects of 2.5–10 µM sorafenib (a multi-tyrosine kinase inhibitor approved for the therapy of advanced HCC): Glo1 ↑, MGO ↑.	Glo1 silencing effect: proliferation ↓ migration ↓ invasiveness ↓	[111] 2019
Human HCC cell line HepG2 incubated with palmitic or oleic acids for 24 h.	Glo1 ↓ in oleic acid treated HepG2. MGO ↑ in both palmitic and oleic acids treated HepG2 and their culture media.	FAs effect: MGO ↑ Glo1 ↓	[106] 2018

↑, increased levels; ↓, decreased levels; =, no change.

### 4.2. MGO in Liver Cirrhosis

Liver tissue is composed of hepatocytes (parenchymal cells) and nonparenchymal cells which include Kupffer cells, hepatic stellate cells, and liver sinusoidal endothelial cells (LSECs). Liver cells which are mostly involved in fibrosis and cirrhosis development are HSCs comprising 5–8% of normal liver cells [113,114]. Oxidative and inflammatory processes coupled with the production of TNF-α and IL-6 observed in the early stages of MASLD lead to the activation of KCs which further activate HSCs. Subsequently, HSCs transdifferentiate into myofibroblast-like cells (through TGF-β-dependent mechanisms) that produce the extracellular matrix (ECM) components including collagen [113,114]. These processes are mediated by signaling pathways leading to cell growth, proliferation, differentiation, and contractility (via MAPKs and rho kinases), as well as proinflammatory routes activation (through NF-κB) [108,113]. Also, LSECs are affected by pathological events and further accelerate them, which leads to enhanced vasoconstriction and portal vein hypertension [113]. A commonly used marker reflecting HSC activation is alpha-smooth muscle actin (α-SMA) expression [114]. In liver cirrhosis, two phases can be distinguished: compensated (where basic liver functions are still sustained, often proceeding asymptomatically) and decompensated (accompanied by ascites, gastrointestinal hemorrhages, and hepatic encephalopathy). Inflammatory processes can lead to the transition from the compensated to the decompensated stage [112].

MGO and Glo1 have been implicated in the development of liver fibrosis and cirrhosis as shown in experiments on rats with induced cirrhosis as well as liver-derived cells in culture [108] (Table 1B). Downregulation of Glo1 (at mRNA and protein levels) positively correlated with the severity of cirrhosis, and associated with the increase in MGO, was reported both in the whole liver and liver cells obtained from cirrhotic animals. Decreased Glo1 expression was noted in cirrhotic hepatocytes, LSECs, as well as HSCs (in which additional Glo1 activity was decreased). However, the inhibition of Glo1 activity with the application of its inhibitors ameliorated cirrhosis features in animal livers, as well as reduced the activation of (LPS-induced) stellate cells in vitro. Similar attenuating effects were observed when LPS-activated HSCs were exposed to 0.1–10 mM MGO. Both Glo1 inhibition and MGO exposition resulted in the reduced release of the inflammatory marker (TNF-α) and fibrotic markers (α-SMA and collagen-I) by HSCs [108] (Table 1B). In human serum, MGO levels reflected the progression of liver cirrhosis being higher in the decompensated stage than in the compensated stage and correlated with liver dysfunction indices [112]. Especially high values were noted in patients with ascites. It has also been observed that the elevation of MGO was correlated with proinflammatory cytokines such as IL-6. Additionally, the authors demonstrated that the concentrations of a different dicarbonyl compound—glyoxal (GO) did not reflect the severity of the disease. Glyoxal is metabolized by a different route than MGO. Therefore, although no direct determinations were conducted concerning the glyoxalase system in the study, the authors implied that the elevation of MGO observed alongside the progression of cirrhosis may be due to the downregulation of this system.

Unfortunately, the above data are insufficient to allow for the elucidation of Glo1 and MGO involvement in liver cirrhosis development. Therefore, more research should be conducted to draw sound conclusions. On the one hand, Glo1 expression decreases during the development of cirrhosis and leads to the elevation of MGO and further consequences yielding AGE formation and RAGE induction which triggers pro-oxidative and proinflammatory pathways through HSCs (and possibly Kupffer cells) activation. These routes further augment profibrotic events such as an excessive generation of ECM components. On the other hand, attenuation of Glo1 activity/MGO elevation can compromise HSC activation—the phenomenon responsible for the acceleration of fibrotic events. It might be hypothesized that in the initial stages of MASLD at low oxidative stress and subtle inflammatory processes, MGO might work as a signaling molecule triggering healing processes, and only when its level overcomes a certain threshold, then it accelerates pathological events. Such hormetic effects of MGO have been previously suggested [67,115,116,117]. Nevertheless, due to a shortage of available data, such suggestions are only of a hypothetical value. The ambiguity of MGO participation in liver cirrhosis, as shown in Figure 4B.

### 4.3. MGO in Liver Cancer

MGO seems to show anticancer activity against hepatocellular carcinoma (HCC) compromising cancer cell adhesion, migration, and invasion [109] (Table 1C). Such conclusions come from experiments on HCC cell lines exposed to MGO [109], as well as the observations on Glo1 expression in human liver cancer tissue which is upregulated in comparison with non-tumorous or cirrhotic tissue [110,111,118,119]. Proliferation of human HCC cell lines was inhibited by Glo1 silencing coupled with MGO accumulation [110] (Table 1C). Therefore, the authors pointed to Glo1 inhibitors as potential medicines in HCC therapy [110]. Several compounds which inhibit Glo1 activity have actually shown promising properties ameliorating HCC [111,120] (Table 1C). Michel et al. [111] reported anti-proliferatory and anti-migratory effects of Glo1 inhibition in HCC cells. Additionally, they observed downregulation of some signaling components involved in pathways promoting cancer growth and metastasis. However, an upregulation of the Nrf2 transcription factor implicated in triggering protective mechanisms in oxidative stress conditions was also noted (Table 1C).

The overexpression of Glo1 observed in HCC and other cancers is attributed to enhanced anaerobic glycolysis typical for cancer cells. An excessive glycolytic flux generates higher amounts of MGO (and D-lactate), therefore cancer cells produce more Glo1 to compromise MGO toxicity [121]. Hence, Glo1 inhibition followed by MGO accumulation can enhance MGO anticancer effects in HCC.

Nevertheless, there are studies on other cancer types which show tumor-promoting effects exerted by MGO. Such observations have been reported (on breast and glioblastoma cancer cells) by Nokin et al. [122], who found that low doses of MGO promoted cancer growth, whereas higher MGO levels caused a reduction in tumor volume. Therefore, as discussed by Bellier et al. [123], a dual impact of MGO on cancer is possible; lower MGO levels might be responsible for the adaptation of cancer cells to carbonyl stress increasing their survival rates (due to the hormetic effect), whereas at higher concentrations MGO exerts toxic effects stimulating apoptotic death of cancer cells.

Therefore, although the above-mentioned findings highlight MGO as a potential anticancer agent in HCC therapy, more experiments should be conducted, since the available data are mainly based on in vitro cell lines experiments. MGO effects on HCC cell lines are summarized in Figure 4C.

## 5. Contribution of Fructose-Derived MGO to MASLD Development

In light of the mounting body of evidence, Fru is a central factor contributing to MASLD. Excess Fru derived both from exogenous (HFCS and other refined sugars foodstuffs) and endogenous (Glc via polyol pathway) sources, promotes lipogenesis and inhibits FAs β-oxidation, thus stimulating steatosis. Additionally, Fru enhances prooxidative and proinflammatory processes which are mostly mediated by uric acid actions [124]. Moreover, other multifaceted Fru effects at the systemic level contribute to the development of obesity, MetS, IR, T2DM, cardiovascular complications, and hypertension [125,126]. These disorders coexist or increase the risk of MASLD development. Considering all these various Fru effects observed nowadays, which result from overnutrition due to an excessive sugary diet, Johnson et al. [126] proposed a fructose survival hypothesis. They proposed that during ages of evolution, human predecessors adapted to the periods of famine, switching their metabolism in advance of crisis into the pathways promoting fat accumulation and lowering energy expenditure. Such organisms feeding on natural sources of Fru (fruits and honey) would accumulate energy which would later allow them to survive in scarcity times. This adaptation might have been favorable in times when the periods of sufficient food sources were interrupted by food depletions. Currently, due to the overconsumption of foodstuffs enriched with HFCS and sucrose, these evolutionarily conserved metabolic pathways become overstimulated and persevere contributing to many obesity-related dysfunctions. Supporting their theory, the authors summarize Fru effects which, although beneficial in the past, now fuel pathological routes. At the systemic level, Fru overload leads to the induction of leptin and insulin resistance. Leptin resistance attenuates a satiety feeling, which stimulates the necessity for food and water intake. Insulin resistance downregulates glucose transporters in the skeletal muscle and adipose tissue (diminishing Glc uptake by these organs), thus saving blood plasma Glc for the brain. Additionally, Fru and IR promote lipogenesis and glycogen synthesis (simultaneously impairing FAs β-oxidation) in the liver. Generally, Fru lowers the metabolic rate through diminishing oxygen consumption (via the reduction in mitochondrial oxidative phosphorylation and stimulation of anaerobic glycolysis). These and other Fru-mediated effects (like blood pressure increase and immune system activation) seem to have improved survival chances in critical conditions, whereas now they contribute to the development of metabolic disorders [126]. As a consequence of excessive Fru phosphorylation by fructokinase C in the liver, ATP depletion coupled with uric acid generation is observed. Although uric acid normally acts as an antioxidant, when overproduced, it mediates many of Fru deleterious events. Additionally, downstream reactions of the fructolytic pathway are associated with the generation of intermediates involved in pathological events, including trioses and their derivatives such as MGO and GA. According to the Brownlee and Giacco [127,128,129] hypothesis, enhanced Glc/Fru oxidative metabolism leads to ROS overproduction which (due to DNA damages) activates poly(ADP-ribose) polymerase (PARP)—an enzyme which further inhibits glyceraldehyde-3-phosphate dehydrogenase (GAPDH). Finally, the glycolytic/fructolytic pathway becomes obstructed at the trioses level resulting in the acceleration of upstream reactions. This causes the accumulation of MGO, GA, and DAGs and the induction of prooxidative and proinflammatory routes. Additionally, MGO can inhibit GAPDH (through the modification of the catalytic cysteine residues) [130]; hence, the accumulation of both reactive carbonyls and ROS can accelerate their own generation in a vicious cycle mode. GA has been demonstrated to exert similar effects as compared with MGO, modifying macromolecules (yielding GA-derived AGEs) and inducing pathological events, thus stimulating MASLD [131]. In light of Takeuchi et al. [131,132,133,134] and Sakasai-Sakai et al. [55,135] observations, (Fru/Glc-derived) GA can exert a major cytotoxic impact mediated by GA-AGE formation, in regard to MASLD and other lifestyle-related diseases onset and development. However, both MGO and GA can modify proteins in a similar mode; interacting with the amino group, Lys, and guanidino group, Arg, and yield some common AGEs (e.g., MG-H1 and ArgP resulting from the action of both molecules) [136]. Therefore, analogical signaling pathways can be triggered by the AGE/RAGE axis following the generation of either of these molecules. Except for the induction of ROS, NF-κB, and other routes, direct intracellular effects through functional protein modifications can contribute to the dysregulation of cellular homeostasis. An example is the proposed impact of MGO (and, according to Takeuchi findings, also GA) on AMPK Arg residues which can impair this enzyme’s susceptibility to regulation by the energy status. As hypothesized by Gugliucci [96,137], uncontrollable dietary Fru influx to the liver and its immediate phosphorylation should lead to the activation of AMPK by elevated AMP (due to ATP depletion). However, the opposite phenomenon has been observed as a consequence of an abundance of this sugar in the liver. Therefore, it is possible that MGO, excessively produced in fructolysis, modifies Arg residues in AMPK. Three Arg side chains are involved in AMPK regulation by AMP. Hence, their modification would impair proper AMPK functioning leading to its downregulation. However, since both MGO and GA are produced from Fru, and both can modify Arg-yielding hydroimidazolone AGEs (MG-H1), it might be suggested that the final effect stems from both molecules’ actions.

When compared to alcoholic liver disease (ALD), MASLD shows analogical pathological stages which include steatosis, hepatitis, fibrosis, cirrhosis, and HCC, conditioned by similar processes comprising lipotoxicity, mitochondrial dysfunction, oxidative stress, ER stress, inflammation, apoptotic cell death, and intestinal dysbiosis [131,138]. As mentioned earlier, both Fru and ethanol seem to trigger similar pathological routes in the liver [39]. In the major pathway of ethanol metabolism (catalyzed by alcohol and aldehyde dehydrogenases and yielding acetaldehyde and acetic acid, respectively) NADH is generated which leads to an increase in NADH/NAD^+^ and ATP/AMP ratios. This causes the inhibition of catabolic processes (TCA and FAs β-oxidation) and promotion of lipogenesis resulting in steatosis and TAGs export to the circulation (leading to hypertriglyceridemia). An important mechanism involved in these disturbances is the inhibition of AMPK due to AMP decline [139]. However, a decrease in AMPK activity observed in ALD might also be a consequence of this enzyme’s functional Arg residue modifications. In contrast, in MASLD the glycating agents might be MGO and GA, and in ALD it could be ethanol-derived acetaldehyde (AA) intermediate—a highly toxic (and accounted to carcinogens) molecule. Therefore, potentially these are highly reactive carbonyl intermediates in the metabolism of Fru/Glc, lipids, and ethanol which contribute to the dysregulation of metabolic homeostasis. Similarly to MGO and GA, AA can interact with amino/guanidino groups of proteins forming the respective AGEs (AA-AGEs) which can impair the structure and function of macromolecules as well as induce RAGE stimulating oxidative stress [131]. Hence, multiple pathological routes impair the liver condition with further consequences including MASLD and ALD development, especially in individuals overusing sweet alcoholic beverages.

Due to the complexity of pathogenic factors underlying MASLD, the development of a therapy that could be efficient in this disorder treatment is a problematic issue. Different therapeutic approaches, focused on a variety of targets are discussed below. Moreover, the potential usefulness of molecules that are able to scavenge MGO, thus ameliorating MASLD symptoms, is further addressed.

## 6. Approved and Potential Therapies in MASLD

**Figure 5:**
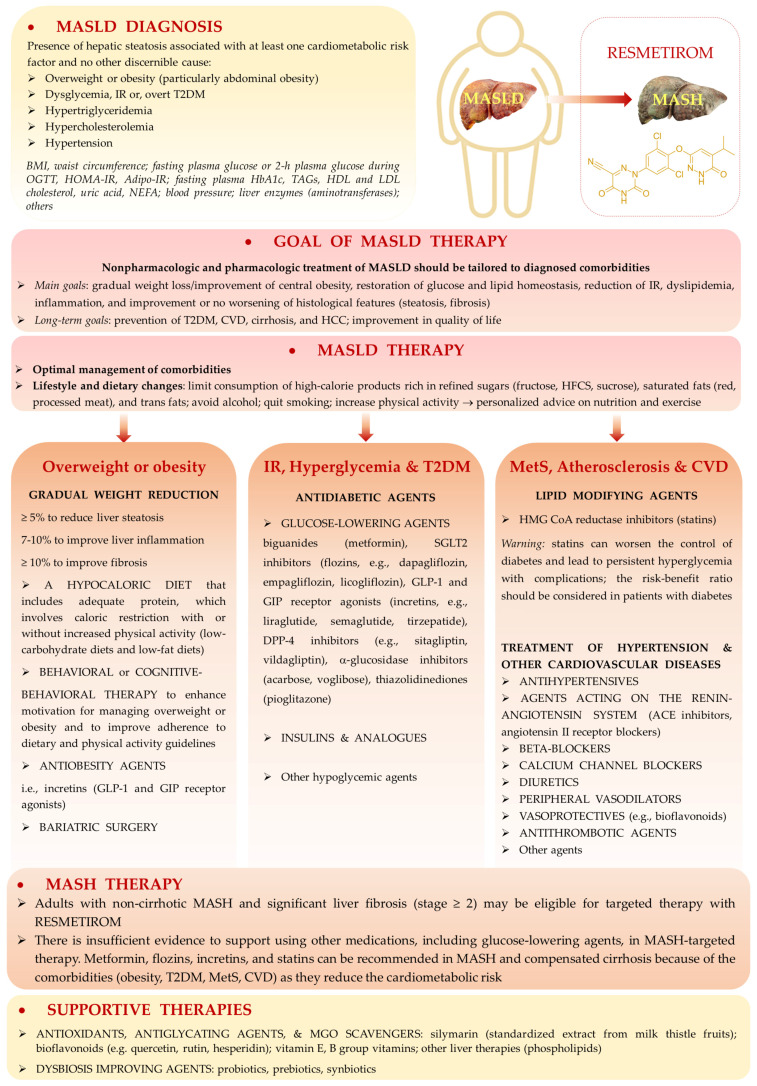
General information on diagnosis and treatment of MASLD. Abbreviations: Adipo-IR, adipose tissue insulin resistance; BMI, body mass index; DPP-4, dipeptidyl peptidase-4; GIP, glucose-dependent insulinotropic polypeptide; GLP-1, glucagon-like peptide-1; HbA1c, glycated hemoglobin; HDL, high-density lipoprotein; HFCS, high-fructose corn syrup; HMG CoA reductase, 3-hydroxy-3-methyl-glutaryl-coenzyme A reductase; HOMA-IR, homeostatic model assessment of insulin resistance; IR, insulin resistance; LDL, low-density lipoprotein; MetS, metabolic syndrome; NEFAs, non-esterified fatty acids; OGTT, oral glucose tolerance test; SGLT2, sodium-glucose co-transporter 2; TAGs, triacylglycerols; T2DM, type 2 diabetes mellitus.

Guidelines for the clinical practice, diagnosis, and treatment of patients with steatotic liver disease have been jointly developed by the European Association for the Study of the Liver (EASL), the European Association for the Study of Diabetes (EASD), and the European Association for the Study of Obesity (EASO) [2]. It states that the diagnosis of MASLD is established in an individual who has documented hepatic steatosis alongside at least one cardiometabolic risk factor that reflects the impact of an abnormal carbohydrate and lipid metabolism. These factors include overweight or obesity (BMI), dysglycemia or T2DM, hypertriglyceridemia, hypercholesterolemia, and hypertension (Figure 5). They may also include peripheral insulin resistance (HOMA-IR, hyperinsulinemic-euglycemic clamp test), adipose tissue resistance to insulin (Adipo-IR, adipose tissue insulin resistance index) [140], and hyperuricemia [141,142]. IR has also been further identified as a distinct risk factor for cardiovascular events, even in non-diabetic patients [143]. Under conditions of insulin resistance, a primary contributor to TAGs and cholesterol accumulation appears to be the overproduction of VLDL, which are metabolized to VLDL remnants, intermediate-density lipoproteins (IDL), and low-density lipoproteins (LDL) [144].

Pharmacological therapies for MASLD are the subject of numerous preclinical and clinical studies. Several groups of therapeutic agents are currently under investigation, including antihyperglycemic agents that increase insulin sensitivity (biguanides, thiazolidinediones) and stimulate insulin secretion (GLP-1 and GIP receptor agonists, DPP-4 inhibitors), SGLT2 inhibitors (flozins), bile acid agonists (ursodeoxycholic acid), FXR agonists (obeticholic acid), peroxisome proliferator-activated receptor/PPAR agonists (e.g., lanifibranor, pioglitazone, and saroglitazar), thyroid hormone receptor β/THR-β agonists (resmetirom), fibroblast growth factor 21/FGF21 analogs (efruxifermin), free fatty acid receptor 4/FFAR4 agonists (omacor), substances that restore AMPK activity (AMPK activators such as metformin), antioxidants (silymarin and vitamin E) [28,145,146,147], gut microbiota modulators (e.g., agents regulating the production of selected SCFAs and bile acid metabolism), and others [148]. However, it should be noted that although there are antidiabetic agents that can improve IR, there are no approved medications specifically designed to treat IR in liver disease. Unfortunately, no specific agent targeting MASLD has been developed to date, especially in the simple stage of steatosis. Nevertheless, there are several promising candidates in controlled clinical trials (RCTs) that improve insulin sensitivity, glucose, and lipid homeostasis and reduce inflammation and progressive liver fibrosis, such as obeticholic acid, elafibranor, cenicriviroc, selonsertib, and resmetirom [149]. Ongoing phase III clinical trials of the above molecules have evaluated their effect on improving liver histology, defined as the resolution of MASH without worsening fibrosis [149,150,151,152]. However, selonsertib monotherapy did not show anti-fibrotic effects in patients with bridging fibrosis or compensated cirrhosis due to MASH [153]. The efficacy of cenicriviroc in treating histologically proven hepatic fibrosis in adults with MASH has also not been confirmed [154]. In contrast, elafibranor was approved by the U.S. Food and Drug Administration (FDA) for treating primary biliary cholangitis in adults based on its ability to reduce alkaline phosphatase (ALP) levels. It has also received positive regulatory approval from the EU [155]. The only FDA-approved small molecule in clinical development for non-cirrhotic MASH with moderate to advanced liver fibrosis is resmetirom. Resmetirom (Figure 5) is a partial THR-β agonist that has shown beneficial effects towards lipid parameters in clinical trials. Compared to the placebo, resmetirom significantly reduced LDL and non-HDL cholesterol, apolipoprotein B (ApoB), hepatic steatosis, and liver stiffness [156,157].

### 6.1. Recommended Therapies and Medications

It appears that the optimal treatment for reducing the risk of progression or reversing the course of MASLD should not target a single risk factor but multiple, often co-occurring and causally related factors. Given the multifactorial pathogenesis of MASLD, combination therapies aiming at more than one therapeutic target may be a rational approach. Obesity commonly coexists with carbohydrate and lipid metabolism abnormalities and elevated blood pressure, but it is also a reversible cause of their development if treated appropriately. Increasing obesity and progressive metabolic disorders lead not only to the development of MASLD but also to other conditions, such as MetS and T2DM, which further increase the risk of CVD. For this reason, MASLD therapy should be personalized and include non-pharmacologic and pharmacologic management appropriate for comorbidities, and patients should remain under multispecialty care. Taking into account the above information, the overall goal of treatment becomes gradual weight loss and restoration of glucose and lipid homeostasis (reduction in IR and dyslipidemia), as well as reduction in inflammation and achieving improvement or no worsening of histological features (improvement/stabilization of steatosis/fibrosis), which may consequently prevent disease progression and even reverse disease symptoms. Therefore, treatment approaches should be tailored to the individual’s needs.

MASLD has a slow onset, is usually asymptomatic, and has metabolic causes. The disease is most commonly diagnosed in patients with obesity or T2DM, but it also affects lean patients. Steatotic liver disease occurs in patients who consume small amounts of alcohol (MASLD < 20–30 g/day), moderate amounts of alcohol (20–30 g/day < MetALD > 50–60 g/day), and in alcohol abusers (ALD > 50–60 g/day). Harmful alcohol consumption is known to accelerate the progression of liver disease in patients with MASLD and chronic hepatitis B and to contribute to the development of cirrhosis or HCC [2]. Obesity, metabolic syndrome, and diabetes also increase the risk of advanced liver disease in individuals who abuse alcohol. Considering the medical recommendations for ALD, the most effective therapy to alleviate the clinical course of the disease and even reverse liver damage is long-term abstinence from alcohol [158]. Therefore, it seems justified that the MASLD approach of limiting excessive consumption of high-calorie products rich in refined sugars (fructose, HFCS, sucrose) and saturated fats, and excluding alcohol will produce relevant positive change [35,38,159]. Indeed, current medical recommendations [2,160,161] refer to changes in dietary habits (limiting the consumption of beverages and foods containing refined sugars, saturated fats, trans fat, alcohol, and stimulants) and lifestyle in the broad sense, including increased physical activity. Thus, the introduction of a hypocaloric diet (caloric restriction, with or without increased physical activity) with adequate protein content and the counteraction of overweight and obesity by gradual weight reduction (by ≥5% to reduce hepatic steatosis, 7–10% to reduce hepatic inflammation, ≥10% to limit fibrosis) is the only recognized treatment strategy for MASLD, that positively affects all biochemical parameters, liver enzymes, steatosis, inflammation, and fibrosis, as well as IR, dyslipidemia, and comorbidities [2,160,161]. Weight loss improves glycemic control, lipid profile, and blood pressure and reduces the risk of T2DM and CVD. However, only a few patients can achieve and maintain weight loss. Therefore, behavioral or cognitive behavioral therapy should be incorporated to overcome this problem, increase motivation for treating overweight or obesity, and improve adherence to dietary and physical activity guidelines [162]. In the case of patients with MASLD and regular body weight, there is no evidence of the beneficial effects related to a hypocaloric diet on liver histology, fibrosis, and clinical liver-related outcomes. For these individuals, health benefits may be achieved by reducing the consumption of refined sugars, especially sweetened beverages, quitting smoking, and avoiding alcohol. Increasing physical activity and reducing visceral fat are also beneficial. Nevertheless, simple lifestyle changes are unlikely to cure advanced stages of MASLD. Therefore, pharmacologic support is needed [2,28,145,163].

In addition to lifestyle interventions, there are established medications to reduce the risk of comorbidities associated with MASLD, such as obesity, MetS, T2DM, and CVD: anti-obesity agents (i.e., peripherally or centrally acting agents), lipid-modifying agents (statins, fibrates, and bile acid sequestrants), diabetes medications (glucose-lowering agents), antihypertensives, diuretics, peripheral vasodilators, beta-blockers, calcium channel blockers, and others (Figure 5). Based on the available evidence, optimal management of comorbidities is recommended, including incretin-based therapies (e.g., semaglutide, tirzepatide) in patients with T2DM or obesity when indicated [2,164,165]. In adults with non-cirrhotic MASH and significant liver fibrosis (stage ≥ 2), targeted therapy with resmetirom (an oral agent) may be used [156,157]. Unfortunately, no pharmacotherapy can target MASH at the stage of cirrhosis [166,167]. There is also insufficient evidence to support the use of any other class of medications, including antihyperglycemic agents, for the treatment of steatohepatitis and liver fibrosis. Therefore, GLP-1 and GIP receptor agonists (liraglutide, semaglutide, and tirzepatide), SGLT2 inhibitors (flozins), thiazolidinediones (pioglitazone), and metformin cannot be recommended in MASH-targeted therapy. These agents should be applied as indicated in patients with MASH and compensated cirrhosis, i.e., in co-existing obesity, T2DM, CVD, and chronic kidney disease because they reduce cardiometabolic risk. They are also safe in MASLD. Unfortunately, there is no definitive evidence that metformin can improve histology in MASH. However, data from observational studies in patients with T2DM and advanced fibrosis or cirrhosis associated with MASH indicate that metformin lowers ALT levels and sensitizes insulin. It regrettably does not significantly improve steatosis, inflammation, or fibrosis in patients with MASLD. There is also evidence that metformin may have a protective effect against HCC. Therefore, metformin is recommended for patients with compensated cirrhosis and preserved renal function [2,167].

According to recommendations [2], statins may be used to reduce cardiovascular events in patients with chronic liver disease, including compensated cirrhosis. However, clinical, observational, and animal studies suggest that some statins (including atorvastatin, simvastatin, and rosuvastatin) may be associated with a small but statistically significant increase in the risk of diabetes, particularly in individuals with IR and prediabetes, despite lowering the LDL cholesterol and improving endothelial dysfunction. This effect was confirmed in a retrospective cohort study in which statin use was associated with the progression to diabetes, significant hyperglycemia, acute glycemic complications, and the need for glucose-lowering medications [168,169]. The exact mechanisms by which statins increase the risk of T2DM are not fully understood. However, evidence suggests that statins may contribute to peripheral IR and pancreatic β-cell dysfunction [169,170,171]. In an animal model, statin treatment was associated with worsening hepatic glycemic control [172]. Therefore, the effect of statins on glucose metabolism and the risk–benefit ratio should be considered in patients with diabetes [168]. Furthermore, other lipid-lowering medications may be prescribed to control TAGs and cholesterol levels, which could help reduce lipid accumulation in the liver [146].

Another liver enzyme-lowering agent is silymarin (standardized extract from milk thistle fruits), although its clinical studies have not shown significant improvement in liver conditions [2,173,174].

### 6.2. MGO, AGEs, and Gut Microbiota as Therapeutic Targets

The etiology of hepatic steatosis in different patients may be related to the co-occurrence of multiple overlapping pathogenic factors (“multiple-hit hypothesis”) resulting from dietary habits, addictions, physical activity levels, and genetic predisposition. Their impact on the risk of MASLD has not been definitively established. Lifestyle factors can be modified by eliminating unfavorable health behaviors, but it is not known how these modifications affect epigenetic changes related to energy metabolism disorders. In a word, it is unclear whether there is a chance to completely reverse the damage inflicted and restore, as far as possible, regular carbohydrate and lipid metabolism in all crucial organs and tissues because of the induction of the metabolic memory phenomenon [175].

Potential therapeutic targets in the course of MASLD are located both in the liver tissue (hepatocytes, Kupffer cells, and hepatic stellate cells) as well as in extrahepatic tissues in WAT (adipocytes) and skeletal muscles (myocytes) (Figure 1). However, much attention has been recently paid to the role of the microbiota and the gut–liver axis, as well as gut dysbiosis (an imbalance in the microbial community). The largest habitat of microbial communities in our body is the colon. Given the direct connection between the gut and the liver, gut dysbiosis is thought to be a factor that affects energy metabolism, which may contribute to the development of metabolic and cardiovascular diseases [26,28,29,30,31].

In individuals with MASLD and obesity, a low-calorie dietary intervention reduces body weight and intrahepatic lipid content [176]. Diet is also considered the most important factor shaping the microbiota ecosystem. Given the extreme complexity of the interaction of dietary components with the profile and function of the gut microbiome, there is still insufficient data to understand these relationships. Nevertheless, although the mechanisms are not fully explained, some dietary approaches are recommended to improve metabolism. For example, a low-carbohydrate diet significantly ameliorates the *Firmicutes/Bacteroidetes* ratio and metabolic markers of dyslipidemia [177]. A Mediterranean diet rich in fiber and phytochemicals can be beneficial because it increases the production of SCFAs (propionate and butyrate) and stabilizes the integrity of the intestinal barrier [178]. A balanced plant-based diet that includes a variety of high-fiber and phytochemical-rich vegetables and fruits has also been shown to enhance gut bacterial diversity and its metabolic activity, including levels of SCFAs [179,180]. Compared with a healthy omnivorous diet, these types of nutrition provide significant cardiometabolic protection [181]. On the contrary, introducing traditional foods rich in fiber and carbohydrates raises the levels of Firmicutes and Prevotella, while increasing foods rich in fiber and animal protein boosts the levels of Bacteroidetes [182]. Similarly, higher levels of physical activity and protein intake correlate positively with microbiome diversity [183]. Therefore, lifestyle changes, especially dietary modifications under the influence of personalized nutritional counseling, may restore the balance of the gut microbiota and be an efficient therapeutic approach to reduce cardiometabolic risk (Figure 5) [30,31,32,184,185,186].

Many natural and synthetic substances (e.g., some pharmaceuticals) have been identified that are harmful to the liver. The development of ALD is induced by cytotoxic ethanol and its intermediate metabolite acetaldehyde, which is characterized by higher reactivity and harmfulness [138,187]. In the case of MASLD, these are probably RCS, the compounds with a similar chemical structure, of which MGO is the major contributor. Methylglyoxal is formed as a by-product of the metabolism of glucose, fructose, lipids, and some amino acids (threonine and glycine) (Figure 2) [84]. It has been found in processed foods (root beer, apple juice, cola, honey, coffee beverages, etc.) [188], although this source is probably of minor importance. A more relevant source of MGO seems to be bacteria inhabiting the gastrointestinal tract that metabolize carbohydrates non-absorbed in the upper part of the GIT [189,190]. Large amounts of MGO are produced by *Proteus mirabilis, P. vulgaris*, and *Morganella morganii*, which have the highest methylglyoxal synthase activity. Relatively high MGO production is also observed in *Lactobacillus, Bifidobacterium*, and *Bacteroides.* Methylglyoxal synthase activity and MGO production are significantly decreased in the presence of SCFAs (propionate and butyrate) and bile acids (cholic acid and deoxycholic acid), confirming that their concentration in the intestine may influence MGO production by colonizing bacteria [191]. Some bacteria have efficient enzymatic systems against MGO, such as GSH-dependent glyoxalase I and II in *E. coli* and bacillithiol/BSH-dependent glyoxalase A and B in *Firmicutes*, that convert MGO to D-lactate. Microorganisms with less efficient protective mechanisms are more sensitive to exogenous MGO, which may lead to quantitative and qualitative changes in the microbiota population under favorable conditions of excessive carbohydrate consumption (e.g., Fru, HFCS, and sucrose) [192]. There is also evidence that the gut microbiota can be considered a source of AGEs. In particular, *E. coli* strains have been shown to release AGEs during growth [193].

Moreover, dietary AGEs (dAGEs) not absorbed in the upper GIT enter the colon, where gut microbiota can metabolize them. That may result in the production of low molecular weight compounds which cross the intestinal barrier. But dAGEs may also contribute to gut dysbiosis, chronic inflammation, and the progression of metabolic disorders by increasing AGE levels in plasma and viscera and altering microbiota-produced metabolites such as SCFAs (decreasing butyrate levels and increasing isobutyrate levels) [194,195,196]. A positive correlation between dAGEs, MG-H1, CML, and steatotic liver disease has been observed in a recent cross-sectional study of the U.S. population. This relationship was mainly found in obese individuals and those with increased waist circumference. Therefore, limiting the intake of dAGEs may be a priority for individuals with obesity to prevent liver disease. Furthermore, restricting dAGE in diet can improve central adiposity, inflammation, and IR in patients with MetS and T2DM [197].

Consequently, we suggest that the strategy targeting MGO and AGEs (MAGEs, dAGEs) could be an additional pharmacological approach for the treatment of diseases associated with impaired carbohydrate and lipid metabolism, like steatotic liver disease. Diminishing MGO and AGE production or scavenging existing MGO/AGEs would result in the amelioration of carbonyl stress, oxidative stress, nonenzymatic glycation, macromolecular cross-linking, and low-grade inflammation. Therapeutics with these properties may also improve microbiota diversity and dysbiosis.

### 6.3. MASLD Therapy with MGO Scavengers and Antiglycation Agents

The antiglycation properties are common to several therapeutic agent groups used in T2DM, MetS, CVD, and inflammation. These include biguanides, thiazolidinediones, sulfonylureas, angiotensin II receptor antagonists, angiotensin-converting enzyme inhibitors, calcium channel antagonists, hydrazinophthalazines, statins, purine derivatives, non-steroidal anti-inflammatory drugs, bioflavonoids, and some B vitamins (e.g., B6 and benfotiamine) [72]. Metformin has been the most extensively studied in this field, and its ability to hinder nonenzymatic glycation may explain pharmacological action beyond the inhibitory effect on hepatic gluconeogenesis. The antiglycation and anti-MGO properties of these therapeutics have been thoroughly discussed in our previous publication [72].

**Figure 6:**
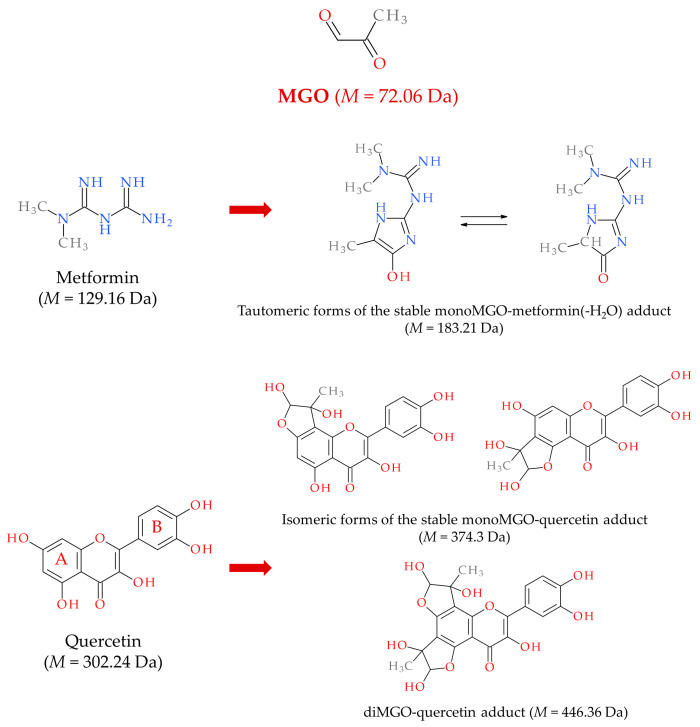
Potential MGO adducts with metformin and quercetin; *M*, molecular mass.

The ability to bind MGO is mainly attributed to metformin [198,199] and various plant flavonoids [200,201,202], including quercetin, taxifolin, luteolin, eriodictyol, hesperetin, phloretin, genistein, and others. The key element of the flavonoid structure that facilitates the uptake of MGO is the phloroglucinol system found in the A-ring [200]. This feature is common among many compounds in this group, including quercetin, taxifolin, and silibinin, which are the primary components of silymarin [173]. However, the structures of flavonoid adducts with MGO are not yet fully understood. Based on spectroscopic studies, Bhuiyan et al. [202] proposed several potential adducts of monoMGO and diMGO with quercetin. Figure 6 illustrates the reported MGO adducts formed with metformin [199] and quercetin [202].

Flavonoids have also been implicated in other health benefits for MASLD patients. These plant-origin compounds reduce postprandial blood glucose levels by decreasing the digestion and absorption of carbohydrates. They achieve this by inhibiting enzymes such as α-amylase and α-glucosidase (e.g., quercetin, taxifolin, luteolin, eriodictyol, genistein, isoquercitrin, hyperoside, silybin, silymarin, and citrus bioflavonoids). Additionally, they inhibit monosaccharide transporters and cotransporters like GLUT2, SGLT1, and SGLT2 (notable examples are phloridzin/phlorizin, curarine, and sophora flavanone G) [203,204,205]. They can also inhibit DPP-4, e.g., quercetin, isoquercitrin, hyperoside, luteolin, and isoliquiritigenin [206]. In addition, many studies show that flavonoids have antioxidant, anti-inflammatory, and anti-atherosclerotic properties [202,207].

Although flavonoid aglycones (non-glycosylated forms) are known to have a higher capacity to trap MGO, their glycosides (glucosides, galactosides, rutinosides, etc.), which are stable in the upper GIT, are subject to degradation in the colon by microbiota, releasing the aglycones and sugar moieties. Thus, they are an additional energy source in this environment. Therefore, flavonoid glycosides with other plant compounds, such as dietary fiber (including plant mucilage, β-glucans, galactomannans, pectins, etc.), are considered prebiotics that interact with the microbiota. This interaction leads to the production of bacterial metabolites from flavonoids, specifically low molecular weight phenols. Released metabolites exert local effects in the colon and help modulate the composition of the resident microorganisms. Once absorbed into the hepatic portal vein, these low-molecular phenols may contribute to liver effects alongside aglycones and their metabolites produced by the host [208]. For instance, DOPAC (3,4-dihydroxyphenylacetic acid), which is generated from quercetin glycosides by the gut microorganism *Flavonifractor* plautii, has been shown to protect cells from the cytotoxic effects of acetaldehyde [209].

The ability to trap MGO and reduce MAGEs in living organisms is also attributed to eriocitrin [210]; it is the citrus fruit flavonoid, and the primary constituent of peppermint leaves. It has been reported that eriocitrin (200 mg/day) lowers glycemia, increases blood GLP-1 levels, reduces the growth of microorganisms associated with gut dysbiosis, and increases the abundance of commensal bacteria in prediabetic patients. As a result, it decreased the growth rate of *Firmicutes* and *Lachnospiraceae*—associated with dysglycemia, *Blautia*—associated with inflammation and altered intestinal permeability, while increasing the abundance of *Ruminococcaceae*—related to the production of SCFAs and anti-inflammatory cytokines. The microbiota changes promoted by eriocitrin have been linked with carbohydrate metabolism and increased GLP-1 production [211].

The best-known hepatoprotective agent, which is used as adjunctive therapy in chronic liver diseases such as MASLD, MetALD, and ALD, is silymarin [212,213]. Silymarin is a complex of flavonolignans isolated from milk thistle fruits (*Silybum mariani fructus*, *Silybum marianum* (L.) Gaertner). Since the components of silymarin are derivatives of taxifolin, featuring a structure that enables covalent binding to MGO (the phloroglucinol motif in the A-ring), we hypothesize that the anti-MGO effect is crucial to its influence on liver steatosis. This action, combined with its anti-glycation, antioxidant, and anti-inflammatory properties, may contribute significantly to the overall effect of silymarin. On the other hand, studies on an animal model of MASLD have shown that silymarin is potent in alleviating IR, primarily by reducing visceral adipose tissue, increasing lipolysis, and inhibiting gluconeogenesis [214]. Velussi et al. [215] confirmed that silymarin (600 mg/day) directly increases insulin sensitivity in diabetic patients, further supporting its therapeutic potential in MASLD. The efficacy and safety of silymarin monotherapy or combined therapy (silymarin + vitamin E + phospholipids or silymarin + simvastatin) in MASLD have been completed by numerous RCTs and several meta-analyses [212,213,216]. In these studies, silymarin, at the standard daily dose, statistically and clinically significantly reduced aminotransferases (ALT, AST), fasting insulin, TAGs, and increased HDL-C levels. In contrast, its effects on GGT, TC, LDL-C, fasting glucose, and HOMA-IR were uncertain. Furthermore, the meta-analysis by Li et al. [213] suggests that silymarin may reduce the fatty liver index (FLI) and improve the hepatic steatosis grade in MASLD patients. Unfortunately, only a few RCTs included pre- and post-treatment liver biopsy data. In a study by Kheong et al. [173], silymarin therapy has diminished liver fibrosis. However, in the RCT conducted by Navarro et al. [174], silymarin has not improved liver histology. Data on the underlying mechanisms of its action, including interaction with the gut microbiota, were provided by Jin et al. [217]. In this RCT, silymarin at 103 mg/day significantly reduced liver stiffness (assessed by FibroScan; Echosens, Paris, France) and ApoB levels compared to the placebo but had no significant effect on other biochemical and non-invasive fibrosis indices. The authors suggested that silymarin may improve liver stiffness by modulating the gut microbiome. Flavonolignans are likely to stimulate the growth of SCFA-producing microorganisms and regulate the metabolism of bile acids. Microbiome analysis revealed an increase in species diversity and enrichment of *Oscillospiraceae*. Using a mouse model of MASLD, Yi et al. [218] showed a decrease in 7-keto-deoxycholic acid and an increase in taurodeoxycholic acid (back to baseline), which restores the negative feedback of the FXR receptor. These findings support that silymarin may modulate the gut microbiome and improve energy metabolism in patients with MASLD.

Other studies suggest that various flavonoids, including those of dietary origin, can significantly improve dyslipidemia (may reduce TAGs, TC, and LDL-C) and liver steatosis. Li et al. [219] have shown that flavonoids alleviate MASLD by beneficial effects on liver function, lipid profile, and inflammation. This systematic review and meta-analysis of RCTs indicated that higher flavonoid intake is associated with a reduced risk of MASLD. The study highlighted the positive effects of flavonoid supplementation (≥500 mg daily) and a significant reduction in ALT (especially for dihydromyricetin), AST (silymarin), GGT (hesperidin), TAGs (genistein), LDL-C (hesperidin and dihydromyricetin), TC, steatosis scores, TNF-α, and NF-κB. However, no significant differences were found for HDL-C, fasting plasma glucose, and HOMA-IR.

The combined effect of two MGO scavengers, metformin and silymarin, on energy metabolism has also been investigated. Hüttl and coworkers [220] evaluated the impact of combining metformin and silymarin on the therapy of hepatic steatosis and dyslipidemia in a rat model with prediabetes. Their findings indicated that this combination therapy was more effective than metformin alone in reducing insulin resistance, promoting weight loss, and improving lipid metabolism. Metformin combined with silymarin significantly lowered serum TAGs and TC while increasing serum HDL-C and decreasing hepatic levels of lipotoxic DAGs. Consistent with other studies, neither metformin monotherapy nor its combination with silymarin altered liver morphology. However, the treatment positively affected the hepatic FA profile and reduced lipid peroxidation markers, attenuating inflammation and oxidative stress.

Therefore, we can assume that metformin, silymarin, quercetin, and certain other flavonoids may benefit the adjunctive therapy of MASLD due to their capacity to bind MGO and inhibit nonenzymatic glycation of macromolecules. These compounds positively influence energy metabolism and liver function (significantly reduce aminotransferases), as well as restore the biodiversity and metabolic activity of the gut microbiota. Finally, they are valuable tools in treatment strategies as they markedly improve IR, glycemic control, and lipid levels.

### 6.4. MGO Scavengers Protect the Activity of AMPK and Promote Autophagy in the Liver

MGO and AGEs have been implicated in insulin resistance and metabolic disorders through several pathogenic mechanisms. These include loss of energy homeostasis, mitochondrial and lysosomal dysfunction, and ER stress. Over the past few years, activation of the AMPK pathway has been identified as a potential therapeutic target for liver disorders [49,53].

Research in experimental models has shown that metformin, silymarin, and quercetin can protect hepatocytes by restoring AMPK activity and enhancing mitophagy [221,222,223,224,225,226,227]. This effect may be coupled to their ability to scavenge MGO and is attributed to protecting the AMPK protein from methylglyoxal-mediated modification. AMPK activation by metformin improves mitochondrial respiration and reduces hyperglycemia in mice fed a high-fat diet [221]. In obese (ob/ob) mice, metformin treatment has been shown to enhance Parkin-dependent mitophagy and reduce ER stress caused by glucotoxicity and lipotoxicity [222]. Similarly, quercetin has alleviated steatotic liver disease in mice fed a high-fat diet by promoting PINK1-Parkin mitophagy [223]. In HCC cells, quercetin has increased the expression of SIRT1, which led to the upregulation of key mitophagy regulators PINK1 and PARK2. This has been accompanied by the colocalization of mitochondria and lysosomes, indicating an enhancement in mitophagy [224]. Silibinin, a primary component of silymarin, has also been found to restore NAD+ levels and activate the SIRT1/AMPK pathway in MASLD models (HepG2 cells and mice) [226]. The compound has also reduced lipid accumulation in HepG2 cells exposed to high-fructose concentrations by inducing autophagy through the AMPK/mTOR signaling pathway [227].

Thus, the above results indicate that metformin, quercetin, silibinin, and silymarin may promote mitophagy by preserving AMPK activity. The above compounds can protect AMPK from nonenzymatic glycation, thereby maintaining its activity. This confirms the potential of metformin and silymarin and their combination as adjunctive therapy in MASLD. However, further molecular studies in experimental models of MASLD are needed to verify the protection of AMPK from MGO-induced modifications.

## 7. Methodology

This review is based on the PRISMA 2020 statement [228] to provide a comprehensive, structured, and transparent approach to collecting and analyzing relevant scientific literature on the current state of knowledge regarding the role of methylglyoxal (MGO) in the development and progression of metabolic dysfunction-associated steatotic liver disease (MASLD, formerly NAFLD) and therapeutic strategies for MASLD that address targets such as insulin resistance, energy metabolism, glucose and fructose metabolism, lipid metabolism, carbonyl stress, oxidative stress, nonenzymatic glycation and advanced glycation end products (AGEs), and chronic low-grade inflammation.

The research question for this review is as follows: What is the role of methylglyoxal in the development and progression of steatotic liver disease associated with metabolic dysfunction, and are there any therapeutic strategies that target the metabolic pathways related to this molecule?

The literature search is as follows: A systematic literature search was conducted to identify relevant studies published in English between 1970 and 2024. The following electronic databases were screened: PubMed, Scopus, Web of Science, and Google Scholar. Keywords used included: “methylglyoxal”, “reactive carbonyl species (RCS)”, “carbonyl stress”, “oxidative stress”, “nonenzymatic glycation”, “advanced glycation end products (AGEs)”, “insulin resistance”, “steatotic liver disease”, “liver steatosis”, “hepatic steatosis”, “hepatosteatosis”, “fatty liver”, “MASLD”, “MAFLD”, “NAFLD”, “steatohepatitis” (“NASH”/”MASH”), “liver cirrhosis”, “liver cancer”, “liver cancer”, “hepatocellular carcinoma”, “energy/glucose/fructose/lipid metabolism in liver/liver diseases”, “steatotic/fatty liver pathology/etiology/pathogenesis”, and “steatotic/fatty liver therapy/treatment”, and combinations of these.

The selection of research is as follows: The article selection process was conducted in two stages—an initial selection of titles and abstracts, followed by full-text evaluation. Two independent reviewers analyzed the titles and abstracts of retrieved articles to identify potentially relevant reports. Pre-defined inclusion and exclusion criteria were used to retrieve the full text of potentially eligible articles.

Inclusion criteria:

Studies published in English between 1970 and 2024.

Studies on methylglyoxal and metabolic dysfunction-associated steatotic liver disease (MASLD/NAFLD).

Studies on carbonyl stress, oxidative stress, nonenzymatic glycation, AGEs, insulin resistance, chronic inflammation, gut dysbiosis, e.g., about their effects on energy/glucose/fructose/lipid metabolism and the etiology and pathogenesis of MASLD/NAFLD.

Exclusion criteria:

Studies published in languages other than English.

Studies not related to the etiology and pathogenesis of MASLD/NAFLD.

Studies not focused on methylglyoxal, reactive carbonyls, carbonyl stress, oxidative stress, nonenzymatic glycation, AGEs, insulin resistance, energy metabolism, glucose metabolism, fructose metabolism, lipid metabolism, the gut–liver axis, or liver disease.

Studies related to the involvement of methylglyoxal in metabolic dysfunctions associated with MASLD (such as obesity, metabolic syndrome, insulin resistance, diabetes, dyslipidemia, atherosclerosis, and hypertension), which were covered in our previous work (see ref. [72]).

Data extraction: Two reviewers independently performed data extraction. The following information was extracted from each study: authors, year of publication, study objectives, study design, research models, statistical methods, areas of implementation, primary findings, and conclusions.

**Figure 7:**
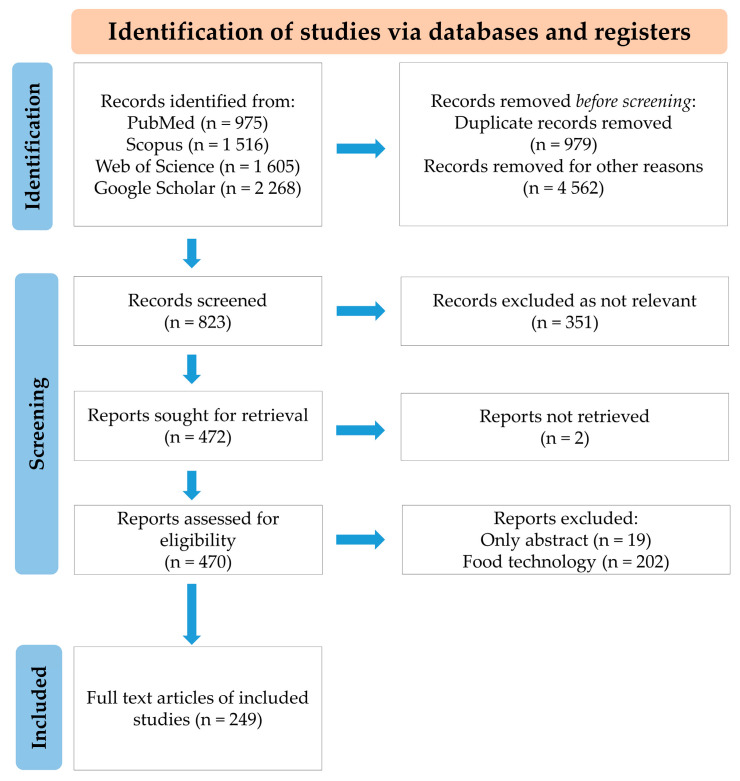
PRISMA flowchart of included studies and registries.

Data synthesis and analysis: The extracted data were thematically organized, analyzed, and synthesized to provide a comprehensive overview of the current knowledge regarding the role of methylglyoxal (MGO) in the development and progression of liver steatosis associated with metabolic dysfunction, as well as therapeutic strategies targeting the involved metabolic pathways. The PRISMA flow diagram of the included studies/registers is presented in Figure 7.

## 8. Conclusions and Remarks for Future Research

**Figure 8:**
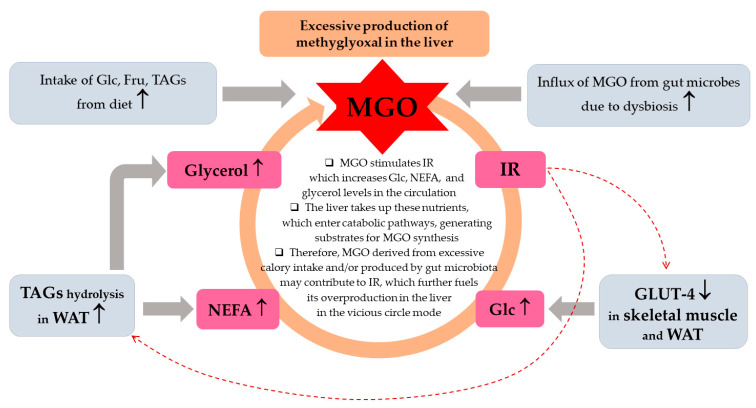
The hypothetical route starting from an excessive production and accumulation of MGO in the liver, and leading to the development of insulin resistance, which further accelerates MGO generation. The pathological processes characteristic for MASLD and its comorbidities and partially mediated by MGO include glycation of structurally and functionally vital macromolecules, which disturbs mitochondria, lysosomes, and endoplasmic reticulum. Subsequently, oxidative stress, proinflammatory, and profibrotic (?) processes are enhanced, which leads to cell death and liver tissue damage. MGO contribution to IR development may be exerted through the modification of insulin, insulin receptor and insulin receptor substrate (as well as other down-stream signaling components). Furthermore, other signal transduction pathways can be impaired due to MGO-modification of their components. A possible intracellular signaling cascade disturbed by MGO can be AMPK. AMPK inhibition leads to the shift from catabolic into anabolic reactions which stimulates lipogenesis and inhibits FAs oxidation. This causes lipid accumulation, contributing to hepatosteatosis. Apart from a direct effect of MGO on macromolecules, it also stimulates prooxidative and proinflammatory pathways through the induction of RAGE.). ↑, upregulation; ↓, downregulation.

An imbalance in calorie intake in favor of high-carbohydrate and high-fat foods leads to metabolic disturbances mediated significantly by methylglyoxal (and other reactive carbonyl species). MGO triggers prooxidative and proinflammatory routes through RAGE induction. Furthermore, MGO may exert its effects via modification of important hormonal and enzymatic targets, such as insulin and its signaling components (causing insulin resistance), AMPK (amplifying anabolic processes), as well as collagen (leading to fibrosis/cirrhosis through ECM dysfunction). Additionally, (M)AGE-associated damages to multiple proteins may be involved in ER stress due to an excessive accumulation of misfolded proteins. MGO possibly affects mitochondrial components contributing to this organellum impairment and oxidative stress. Such disturbances impair cellular homeostasis diverting cell’s machinery toward death through the attenuation of autophagy. Finally, MGO may block glycolysis/fructolysis due to the inhibition of GAPDH which favors the generation of by-products of these processes (Fru, trioses, DAGs, MGO, and GA), thus accelerating detrimental pathways in a vicious-cycle mode. Therefore, via multiple routes, MGO can exacerbate MASLD where it seems to participate in insulin resistance, oxidative stress, inflammatory processes stimulation, and induction of lipogenesis. Since insulin resistance seems to be the central pathological knob in MASLD and its comorbidities, and MGO impact on systemic IR development has been well documented [229,230,231,232,233,234,235,236,237,238,239,240,241,242,243,244], the proposed MGO implication in IR is presented and discussed in Figure 8.

Increasing the accumulation of data has allowed us to fill the gaps in the overall picture of MASLD etiopathogenesis. Nevertheless, more research should be conducted to better elucidate MGO function in this liver disorder. Its detrimental role in the generation of metabolic disturbances leading to the induction of MASLD is well substantiated. However, MGO involvement in the processes associated with the development of fibrosis and cirrhosis as well as liver cancer is not clear. There are only a few studies referring to these issues, and no consistent conclusions can be drawn. As discussed, some data indicate profibrotic, and others antifibrotic actions of MGO. Experiments on MGO effects on hepatocellular carcinoma point to its anticancer actions. However, only several in vitro cell line studies have been performed in this area so far. Therefore, MGO function in early MASLD is well evidenced, but no ultimate conclusions can be drawn on its participation in liver cirrhosis nor in liver cancer. It can be only hypothesized that MGO effects are highly dependent on its quantity. Since MGO is constantly generated at low levels, mainly as a glycolytic by-product, it might be suggested that at low concentrations it stimulates pro-survival processes in the cells. Such actions would activate protective mechanisms to prepare the cell against oxidative stress and inflammation. However, when the level of MGO crosses the threshold of the protective capacity of the cell, its detrimental effects seem to prevail which triggers pathological routes. Such a dual role may be implied from studies on MGO involvement in liver cirrhosis development. A similar phenomenon may be observed in cancer cells which are characterized by increased anaerobic glycolysis associated with augmented MGO generation. Thus, low level MGO (and other glycolytic intermediates and by-products) may promote cancer growth, whereas only at higher concentrations of MGO gains anticancer activity.

Despite the tremendous advances in our knowledge of liver steatosis, many important areas of MASLD require further research to improve its targeted therapy. Thus, there is an urgent need for continued studies in vitro and in vivo on the role of MGO and MAGEs in the etiology and pathogenesis of MASLD, considering the disease stage from simple steatosis to cirrhosis and HCC.

Several small molecules are currently being assessed in clinical trials to identify the most effective MASLD treatment. These agents differ in their mechanism of action by addressing different cellular targets and metabolic pathways or proinflammatory and pro-fibrotic factors. Unfortunately, except for resmetirom approved for non-cirrhotic MASLD therapy, phase III trials have not met expectations. Therefore, weight loss through a hypocaloric diet and lifestyle changes are the only accepted treatments for MASLD in its early stages. Nevertheless, it appears that therapeutics that can capture and bind MGO, thereby reducing levels of cytotoxic methylglyoxal-derived AGEs (MAGEs), have beneficial effects on insulin resistance, dyslipidemia, aminotransferases levels, gut dysbiosis, and chronic low-grade inflammation. Although agents like metformin and silymarin exert modest effects on liver histology, their antiglycation, anti-MGO, antioxidant, anti-inflammatory, AMPK protecting, mitophagy promoting, and gut microbiota-modulating effects could provide additional support for steatotic liver disease therapy. However, to conclusively prove their potential, particularly regarding the mechanism that targets MGO and MAGEs, further well-designed studies in experimental MASLD models and MASLD patients are required. Hence, it is advisable to identify intra- and extracellular MGO targets in the context of steatotic liver development. For example, although AMPK is known to be downregulated in MASLD, and it may be partially caused by MGO modification, no direct evidence has been reported in experimental studies. Additionally, MGO is known to be able to modify collagen, which would impair ECM structure and might accelerate fibrosis. However, such a route, although possible, has not been investigated in a fibrosis model. Therefore, detailed elucidation of MGO-modified macromolecules involved in MASLD pathogenesis is recommended in future studies. Such experiments should start from in vitro experiments on the MGO effect on chosen isolated macromolecules, as well as intracellular organelle (preferably mitochondria) and liver-derived cell lines (hepatocytes, hepatic stellate cells, and Kupffer cells).

Epidemiologic studies suggest a positive correlation between a diet rich in saturated fatty acids and refined sugars (especially Fru) with increased MASLD risk. However, only a few data are available that systematically compare nutritional components, which would elucidate their additive or synergistic effects regarding MASLD onset and development. Thus, experiments aimed at such comparisons should be conducted initially in liver cell lines and then in vivo on animal models. Such studies might focus on experiments evaluating an overload with Fru, Glc, saturated, and trans monounsaturated fatty acids (e.g., elaidic acid) in different combinations. In this way, the effects of single molecules might be compared with the impact of their mixtures on the liver condition.

Most studies associated with MGO involve different MASLD stages performed on cell lines and animal models. In turn, there is a scarcity of data determining MGO and MAGE levels in patients diagnosed with MASLD. Actually, there is only one such report showing the correlation between increased MGO and liver cirrhosis severity. Hence, there is a need for more studies that would assess the concentration of MGO and MAGE in blood plasma as well as in liver specimens in individuals with MASLD confirmed with the most accurate diagnostic methods.

Finally, the search for molecules with the most MGO scavenging potential should be continued. Such compounds, either directly binding and removing MGO or activating the glyoxalase system that metabolizes MGO, would give rise to new medicinal drugs that could be applied in MASLD therapy.

## Data Availability

Not applicable.

## Abbreviations

The following abbreviations are used in this manuscript:

ACC	acetyl-CoA carboxylase
AceCS	acetyl-CoA synthetase
AGEs	advanced glycation end products
AIFM2	factor mitochondria associated 2A
ALP	alkaline phosphatase
ALT	alanine aminotransferase
AMPK	AMP-activated protein kinase
ArgP	Argpyrimidine
ApoE^−/−^	apolipoprotein E knockout
AST	aspartate aminotransferase
ATAUC	adipose tissuearea under the curve
α-SMA	alpha-smooth muscle actin
BCAA	Branched-chain amino acids
CaMKKβ	Ca21/calmodulin-dependent protein kinase kinase β
CCl_4_	carbon tetrachloride
CD43	leukosialin (mucin-like protein expressed on the surface of most hematopoietic cells)
CEdG	*N*^2^-carboxyethyl-20–deoxyguanosine
CEL	*N*^ε^-(1-carboxyethyl)lysine = *N*^6^-(1-carboxyethyl)lysine
Chol	Cholesterol
ChREBP	carbohydrate-responsive element-binding protein
CRP	C-reactive protein
CTGF	connective tissue growth factor
CVD	cardiovascular disease
DAGs	Diacylglycerols
DAMPs	damage-associated molecular patterns
DMT1	divalent metal transporter 1
DNL	de novo lipogenesis
ECM	extracellular matrix
EVsFAs	extracellular vesiclesfatty acids
FAS	fatty acid synthase
Fru	Fructose
FXR	farnesoid X receptor
GA	Glyceraldehyde
GAPDH	glyceraldehyde-3-phosphate dehydrogenase
GGT	γ-glutamyl transpeptidase
GIT	gastrointestinal tract
Glc	Glucose
Glo1	glyoxalase 1
Glo2	glyoxalase 2
GLUT-4	insulin-dependent glucose transporters in skeletal muscle and adipose tissue
GPx	glutathione peroxidase
GPX4	glutathione peroxidase 4
GSH	reduced glutathione
GSSG	oxidized glutathione
HCC	hepatocellular carcinoma
HDL-Chol	high-density lipoproteins cholesterol
Hep G2	epithelial hepatoblastoma cell line
HFCS	high-fructose corn syrup
HFD	high-fat diet
HHTg	hereditary hypertriglyceridemic rats
HIF	hypoxia-inducible factor
HO	heme oxygenase
HOMA	homeostatic model assessment
HSCs	hepatic stellate cells
IR	insulin resistance
IRS-1,2	insulin receptor substrate 1,2
JNK	c-jun NH2-terminal kinase
KCs	Kupffer cells
LKB1	liver kinase B1
LPO	lipid peroxidation
LPS	Lipopolysaccharide
LSECs	liver sinusoidal endothelial cells
MAGEs	MGO-derived advanced glycation end products
MAPKs	mitogen-activated protein kinases
MASLD	metabolic dysfunction-associated fatty liver disease
MCP-1	monocyte chemoattractant protein 1
MDA	Malondialdehyde
MetS	metabolic syndrome
MG-dG	3-(20–deoxyribosyl)-6,7-dihydro-6,7-dihydroxy-6/7-methylimidazo-[2,3-b]purin-9(8)one
MG-H1	*N^δ^*-(5-hydro-5-methyl-4-imidazolon-2-yl)-ornithine
MG-H2	2-amino-5-(2-amino-5-hydro-5-methyl-4- imidazolon-1-yl)-pentanoic acid
MG-H3	2-amino-5-(2-amino-4-hydro-4-methyl-5-imidazolon-1-yl)-pentanoic acid
MGO	Methylglyoxal
MKK7	mitogen-activated protein kinase kinase 7
NEFAs	non-esterified fatty acids
NF-κB	nuclear factor-kB
NOX	NADPH oxidase
Nrf2	nuclear factor erythroid 2-related factor 2
PARP	poly(ADP-ribose) polymerase
PRRs	pattern recognition receptors
PUFAs	polyunsaturated fatty acids
p38 MAPK	p38 mitogen-activated protein kinase
RAGE	advanced glycation end products receptor
RCS	reactive carbonyl species
RCT	randomized controlled trial
RNS	reactive nitrogen species
ROS	reactive oxygen species
SCFAs	short chain fatty acids
SMAD3	a protein involved in TGF-β signal transduction
SOD	superoxide dismutase
SREBP	sterol regulatory element-binding protein
TAGs	Triacylglycerols
TAK1	TGF-β-activated kinase 1
TBARS	thiobarbituric acid reactive substances
TC	total cholesterol
TCA	tricarboxylic acid cycle (Krebs cycle)
T2DM	type 2 diabetes mellitus
TfR1	transferrin receptor 1
TGF-β	transforming growth factor β
THP	Tetrahydropyrimidine
TNFα	tumor necrosis factor alfa
THR-β	thyroid hormone receptor β
Trx	Thioredoxin
WR	Wistar rats
Glossary	
Term	Definition
AMPK	AMP-activated protein kinase is an energy sensor which regulates metabolism, switching the processes between catabolic and anabolic depending on the energetic status of the cell. AMPK requires threonine (Thr^172^) phosphorylation for its activation, which is achieved by three different kinases (liver kinase B1—LKB1, Ca21/calmodulin-dependent protein kinase kinase β—CaMKKβ, and TGF-β-activated kinase 1—TAK1) induced by various signals [48]. AMPK is sustained in this active phosphorylated formed at low energy level by AMP (and ADP) binding, whereas higher ATP concentration inactivates the enzyme. Therefore, at low energy levels reflected by high AMP/ATP ratio, AMPK is active and regulates specific target enzymes, increasing lipid oxidation and mitochondrial biogenesis, whereas the synthesis of lipids and glycogen is inhibited. In such a way, energy-consuming anabolic pathways are attenuated in favor of induced catabolic pathways aimed at the replenishment of energy. One of the important targets of AMPK is acetyl-CoA carboxylase (ACC) which produces malonyl-CoA. Malonyl-CoA is a substrate for palmitic acid synthesis, but also it is an inhibitor of carnitine palmitoyl-transferase 1 (CPT1)—an enzyme involved in the transport of long-chain fatty acids to the mitochondrium for β-oxidation. AMPK phosphorylates and inhibits ACC which leads to a decrease in malonyl-CoA, thus attenuating palmitic acid synthesis. Simultaneously, a drop in malonyl-CoA level releases the inhibition of CPT1 which enables entry of FAs to the mitochondrium for β-oxidation. Consequently, the synthesis of fatty acids (DNL) in the liver decreases, whereas mitochondrial β-oxidation of FAs increases [48,49]. Additionally, AMPK phosphorylates and inhibits transcription factors (sterol regulatory element-binding proteins, SREBPs) responsible for the expression of enzymes involved in FAs, triacylglycerol, and cholesterol synthesis.
Apoptosis	Apoptosis is a type of regulated cell death necessary for the clearance of destroyed cells to maintain tissues in healthy condition. Damaged cells undergo shrinking and membrane blebbing, followed by the removal of macrophages. Apoptosis can be induced by internal or external signals. The intrinsic (mitochondrial) pathway is initiated by receptor-independent signals generated in the cell, such as ROS accumulation. The major regulator detecting DNA damages and deciding on the further cell’s fate is the tumor suppressor p53 protein. This protein controls Bcl-2 family proteins. Bcl-2 and Bcl-XL belong to pro-survival factors, whereas Bax or Bak stimulate apoptotic events. In response to strong proapoptotic signals (e.g., resulting from the accumulation of unrepairable genetic defects), they increase the permeability of the inner mitochondrial membrane and release of proapoptotic factors, including cytochrome C. Extrinsic pathways are induced by death receptors which activate caspase cascade starting from caspase 8. Both intrinsic and extrinsic pathways converge on the execution phase initiated by the activation of caspase 3 [245].
Autophagy	Autophagy (“self-eating”) is a conservative, pro-survival process employed by normal cells to recycle the wastes (defective or unnecessary intracellular structures and macromolecules). It is divided into macroautophagy, selective autophagy, microautophagy, and chaperone-mediated autophagy, from which macroautophagy—the most common and best studied type is usually referred to as simply autophagy [77]. Macroautophagy proceeds with the formation of an autophagosome (a sequestration of a fragment of cytoplasm by surrounding its contents with double membranes) which next fuses with a lysosome yielding autophagolysosome. Lysosomal hydrolases degrade enclosed molecules generating products which are exported back into the cytoplasm where they are reused for the synthesis of new molecules, such as amino acids for novel protein formation. Degradation products also serve as a source of energy (e.g., fatty acids). Autophagy functions constitutively at a low level, but in conditions of nutrients and/or oxygen deprivation as well as ER stress it intensifies to increase the survival odds in stressful conditions. Signaling pathways regulating these events include mammalian targets of rapamycin (mTOR), as well as AMPK routes. mTOR being active at sufficient nutrient satiation suppresses the induction of autophagy, whereas, during starvation, inflowing signals block the mTOR route, which triggers the autodigestion mechanism. In turn, AMPK being the sensor of intracellular energy level, becomes activated at ATP depletion, therefore it induces autophagy during energy exhaustion [246,247].
De novo lipogenesis (DNL)	De novo lipogenesis is the process of converting excess dietary carbohydrates (mainly Glc and Fru) into fatty acids. FAs are produced from acetyl-CoA molecules generated in carbohydrate catabolism (in glycolysis from Glc and fructolysis from Fru) or acetate generated by microbiota Fru fermentation [36,37].
Ferroptosis	Ferroptosis is a type of non-apoptotic regulated cell death stimulated by iron-dependent lipid peroxidation and characterized by cell swelling and plasma membrane rupture. It is promoted under conditions of a high concentration of free iron, ROS, and membrane phospholipids containing polyunsaturated fatty acids (PUFAs). Free iron in the presence of superoxide radicals leads to the generation of hydroxyl radicals which stimulates lipid (PUFAs) peroxidation. This causes degradation of biological membranes and cell death. Therefore, the upregulation of factors which increase iron and ROS accumulation promotes ferroptosis. They include proteins involved in iron transport and generation (e.g., transferrin receptor or heme oxygenase—HO), as well as superoxide production (like NADPH oxidases—NOXs). Ferroptosis can also be promoted by selective autophagy such as ferritinophagy and lipophagy which are associated with the release of iron cations and fatty acids, respectively. In turn, the main mechanism restraining ferroptosis includes glutathione peroxidase 4 (GPX4)—a selenium-dependent antioxidative enzyme which reduces phospholipid hydroperoxides, as well as reduced glutathione—a co-substrate in GPX4 reaction. The sufficient level of GSH in the cell is conditioned by the influx of its precursor—cystine. Therefore, the transporter (x_c_^-^ antiporter) involved in cystine transport to the cells plays an important function here. Another component protecting from ferroptosis is a apoptosis-inducing factor for mitochondria associated 2A (AIFM2/FSP1) protein, which seems to act in two ways. Firstly, having an oxidoreductase activity, AIFM2 reduces coenzyme Q10 to its ubiquinol form, which generates a hydrophobic antioxidant. Secondly, it protects from membrane injury through activating the endosomal sorting complexes required for transport (ESCRT)-III–dependent membrane repair in the plasma membrane [47].
Lipophagy	Lipophagy is a type of selective autophagy controlling lipid metabolism. It proceeds through the surrounding of lipid droplets by a double membrane (autophagosome) which further fuses with the lysosome. Subsequently, lysosomal lipases hydrolyze lipid contents releasing their products, e.g., fatty acids. Fatty acids are directed to the mitochondrium for β-oxidation [248,249].
Mitophagy	Mitophagy is a type of selective autophagy responsible for a steady-state turnover of mitochondria as well as the clearance of injured mitochondria. Damage to mitochondria (reflected by the loss of the mitochondrial membrane potential), induces Parkin (E3 ubiquitin ligase) translocation to mitochondria. Parkin ubiquitinates outer membrane proteins which induce mitophagy. Subsequently, the mitochondrium become surrounded by a double membrane (autophagosome) and fuse with the lysosome whose hydrolases degrade mitochonrium components. Parkin recruitment is mediated by PINK1 [247].
